# Association of postnatal severe acute malnutrition with pancreatic exocrine and endocrine function in children and adults: a systematic review

**DOI:** 10.1017/S0007114522001404

**Published:** 2023-02-28

**Authors:** Farzana Ferdous, Suzanne Filteau, Nanna Buhl Schwartz, Sehlulekile Gumede-Moyo, Sharon Elizabeth Cox

**Affiliations:** 1 School of Tropical Medicine and Global Health, Nagasaki University, Sakamoto Campus, Nagasaki, Japan; 2 Nutrition and Clinical Services Division, International Centre for Diarrhoeal Disease Research, Bangladesh, Dhaka, Bangladesh; 3 Faculty of Epidemiology and Population Health, London School of Hygiene and Tropical Medicine, London, UK; 4 Dept of Nutrition, Sports and Exercise, University of Copenhagen, Frederiksberg, Denmark; 5 Institute of Tropical Medicine, Nagasaki University, Sakamoto Campus, Nagasaki, Japan; 6 UK Health Security Agency, 61 Colindale Avenue, London, UK

**Keywords:** Malnutrition, Diabetes, Pancreas, Exocrine, Endocrine

## Abstract

Severe acute malnutrition may lead both concurrently and subsequently to malabsorption and impaired glucose metabolism from pancreatic dysfunction. We conducted a systematic review to investigate the associations of current and prior postnatal wasting malnutrition with pancreatic endocrine and exocrine functions in humans. We searched PubMed, Google Scholar, Web of Science and reference lists of retrieved articles, limited to articles in English published before 1 February 2022. We included sixty-eight articles, mostly cross-sectional or cohort studies from twenty-nine countries including 592 530 participants, of which 325 998 were from a single study. Many were small clinical studies from decades ago and rated poor quality. Exocrine pancreas function, indicated by duodenal fluid or serum enzymes, or faecal elastase, was generally impaired in malnutrition. Insulin production was usually low in malnourished children and adults. Glucose disappearance during oral and intravenous glucose tolerance tests was variable. Upon treatment of malnutrition, most abnormalities improved but frequently not to control levels. Famine survivors studied decades later showed ongoing impaired glucose tolerance with some evidence of sex differences. The similar findings from anorexia nervosa, famine survivors and poverty- or infection-associated malnutrition in low- and middle-income countries (LMIC) lend credence to results being due to malnutrition itself. Research using large, well-documented cohorts and considering sexes separately, is needed to improve prevention and treatment of exocrine and endocrine pancreas abnormalities in LMIC with a high burden of malnutrition and diabetes.

Wasting malnutrition remains common both for children in low- and middle-income countries (LMIC) and for adults with severe infections, notably HIV or tuberculosis. As treatments for severe acute malnutrition improve^(1)^ and drugs become increasingly available and effective for severe infections, more people survive but the long-term consequences of their malnutrition are not fully understood^(2,3)^. Acute nutritional deficits during the prenatal period affect the structure and function of organs such as the pancreas which have fundamental roles in metabolism^(2,4)^. While there is information from animal models and from human studies of prenatal malnutrition, usually indicated by a proxy of low birth weight^(5)^, the consequences of postnatal undernutrition on human pancreatic structure and function and later chronic disease development are not well documented.

Both pancreatic endocrine (i.e. production of hormones such as insulin or glucagon) and exocrine (i.e. production of enzymes to aid digestion and subsequent nutrient absorption) functions are critical for nutritional metabolism and chronic diseases including diabetes. A previous systematic review of effects of severe acute malnutrition on pancreatic exocrine function in children concluded that there was evidence of association but could not determine causality^(6)^. Diabetes mellitus (DM) is one of the most common non-communicable diseases worldwide and is rapidly increasing, particularly in LMIC^(7)^.While it is established that overweight and obesity in adult life increase the risk of type 2 DM^(8)^, the contribution of prior malnutrition to the aetiology of DM and its potential interaction with later overweight across the global context remains unclear.

In 1965, a WHO Expert Committee reported that ‘the evidence that undernutrition protects adult populations from diabetes seems unassailable’^(9)^. In 1980, they reported that ‘in some societies, malnutrition is probably a major determinant of diabetes’^(10)^. In 1985, malnutrition-related DM was included as a classification category of DM divided into two subtypes, protein deficiency pancreatic diabetes and fibrocalculous pancreatic diabetes, both commonly reported in tropical countries and usually associated with a history of undernutrition^(11)^. This classification has since been dropped and the literature is inconsistent in the terms and diagnostic criteria used for these atypical forms of diabetes. As well as the above classifications, other commonly used terms have included ‘tropical diabetes’, ‘malnutrition-associated diabetes’ and ‘African diabetes’. A recent systematic review concluded that, based on currently limited data, two main phenotypes of atypical diabetes emerge, differing in usual age of onset and in the requirement for lifelong insulin but both occurring in younger ages than is typical for type 2 DM and in underweight individuals or normal weight/modestly overweight individuals; both phenotypes have some features similar to type 1 DM^(12)^. Previous reviews have assessed famine, or malnutrition in a particular age group, and either exocrine function or diabetes as an outcome of endocrine dysfunction but not detailed markers of glucose metabolism^(6,13)^. The present study includes detailed glucose metabolism markers and diabetes as well as exocrine pancreas functions in relation to the less studied, postnatal period of exposure to acute malnutrition, not limited to the postnatal period but including childhood and adulthood and from infection-associated malnutrition. Excluding this, most common type of malnutrition exposure could lead to underestimating the impact on populations which might underlie the large increase in diabetes in populations still experiencing a high burden of infectious diseases. Finally, the decision to include anorexia nervosa (AN) as another exposure was to allow the comparison with malnutrition in which diet restriction, rather than infection, has the main causal role.

This systematic review aims to describe the available evidence to determine if severe acute postnatal malnutrition causes persisting changes in pancreatic endocrine and exocrine function and later increased risk of DM.

## Methods

### Search strategy

An electronic literature search was performed on PubMed, Web of Science and Google Scholar to identify studies published in English from the earliest available date to 1 February 2022. Detailed search terms are shown in Supplementary Data 1. Studies were eligible for inclusion if they reported human pancreatic function in relation to exposure to postnatal malnutrition identified through clinical or anthropometric methods in hospitals, clinics or communities, famine or eating disorders. Studies were excluded if written in languages other than English, if the full text was unavailable, if study participants had no prior or current malnutrition exposure or only prenatal malnutrition exposure or if stunting (chronic malnutrition) without wasting (acute malnutrition) was the exposure. We included cross-sectional studies to investigate acute associations between malnutrition and pancreas function and trials, cohort studies or retrospective case–control studies to investigate longer term outcomes. Case series and case reports and studies with ≤ 10 participants were excluded from the review owing to the high potential for bias. Studies describing abnormal pancreatic function, for example, due to cystic fibrosis, leading to malnutrition were also excluded. Cancer studies were excluded since, although many cancers may result in malnutrition, the added metabolic complications of cancers and their treatment would make it difficult to determine the effects of malnutrition itself. However, we did include studies of malnutrition secondary to serious infections such as HIV or tuberculosis with the rationale that infections are virtually always part of severe malnutrition, including classical malnutrition in young children.

Duplicates were identified and removed, and the titles and abstracts were reviewed to determine possible eligibility by a single reviewer (FF). Additional studies were identified by manually searching the reference lists of included papers and previous reviews or meta-analyses. The full texts of the relevant articles were obtained and independently reviewed for final selection according to the eligibility criteria by at least two of the authors. Any differences in judgment were discussed with all authors to reach consensus.

### Quality assessment

A quality assessment checklist was developed based upon the Strengthening the Reporting of Observational Studies in Epidemiology (STROBE)^(14)^ and Consolidating Reporting of Clinical Trials (CONSORT) checklists^(15)^. The quality assessment checklist comprised sixteen items for cross-sectional studies, nineteen items for cohort studies, twenty items for case–control studies and twenty-two items for clinical trials where scores for each question item ranged from 0 to 2. Scores were assigned as follows: ‘0’ for no information/unlikely or not reported/poor or inappropriate description; ‘1’ for partially or possibly reported/satisfactory and ‘2’ meaningfully reported/good. Using these checklists, three authors (FF, SC, and NBS) independently evaluated the included articles. Scores and decisions were then discussed before assigning an overall quality rating of each study with each article’s quality rated as ‘high’ if score ≥ 80 %; ‘medium’ if score 60–79 %; ‘low’ if score < 45–59 % or very low if score < 45 %.

### Data extraction

Details of the included studies were extracted into an Excel file under the following headings: first author, year of publication, study design, country, quality category, age when participants were malnourished, number of participants, participant inclusion/exclusion criteria, diagnostic criteria or definition of malnutrition used, pancreatic outcomes assessed, a description of any interventions or treatment/nutritional rehabilitation received, the type, timing and frequency of pancreatic function outcome assessments conducted and the main or most salient results plus a column for further comments.

## Results

### Articles included and excluded

**Fig. 1.**
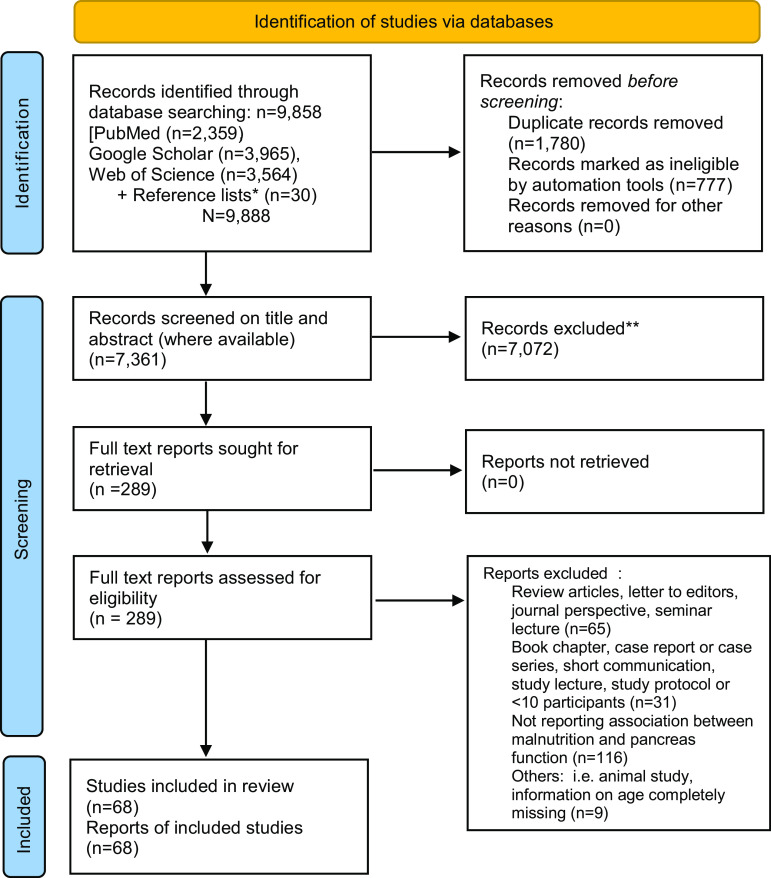
A systematic review flow diagram of the number of articles identified and examined at each stage of the review. *Reference lists from identified full-text articles

After the removal of duplicates, 8108 articles were identified. After screening of abstracts, full texts of 259 papers were obtained (Fig. 1). An additional thirty articles meeting the study inclusion criteria were obtained from a manual screening of article reference lists. Of the 289 articles, 221 articles were excluded due to not meeting the inclusion criteria, leaving sixty-eight articles. The list of excluded studies with reasons is provided in Supplementary Data 2.

The included articles represent results from twenty-nine countries and a total of 592 530 participants. Results are presented divided into three tables based on the nature of the malnutrition exposure and then split into exocrine and endocrine outcomes. Table 1 includes studies reporting the association between malnutrition in young children and exocrine (*n* 13)^(16–28)^, or endocrine (*n* 19)^(29–47)^, pancreatic function either concurrently or after follow-up, most of which was short term. Table 2 includes studies reporting the association between malnutrition in older children or adults, mainly AN patients, and exocrine (*n* 3)^(48–50)^ or endocrine (*n* 15)^(51–65)^ pancreatic function. Table 3 includes studies reporting the association between famine exposure in infancy to young adulthood and glucose metabolism or endocrine (*n* 18)^(66–83)^ pancreatic function; there were no papers describing exocrine pancreas function after childhood famine exposure.

**Table 1.** Association between concurrent/short-term outcome of childhood malnutrition and pancreatic function

Author and reference	Study design	Country/quality assessment	Age when malnourished	No of participants	Inclusion/exclusion criteria	Definitions of MN	Pancreatic function times and tests	Intervention or nutritional rehabilitation (NR)	Main relevant findings	Comments
Exocrine pancreatic function										
Barbezat *et al.*, 1967^(28)^	Clinical cohort	South Africa V. Low	Mean age 18·3 months	• 21 MN inc: • 14 KK • 7 MS • 10 treated for KK 5 years before (5-year FU) 7 non-MN controls	Inclusion unclear • Similar age and population group non-MN controls Exclusion criteria not specified	• KK by clinical criteria • MS: < 61 % expected weight (Boston ref) without oedema	• 1 d after admission and after acute recovery (KK, *n* 11, day not specified) • Duodenal juice volume, pH amylase, lipase, trypsin, chymotrypsin before and after SST	NR: K-Cl daily supplements, after stabilisation, full cream milk, then mixed diet	• Low amylase both basal and after SST in MN but increased after acute recovery • Basal lipase low in only KK, improved on acute recovery • Slightly decreased trypsin in MN which improved on acute recovery • Both basal and after SST chymotrypsin v low in MN; improved after acute recovery but ↓ in 5-year FU group. • Vol/kg and pH not affected by MN	• Unclear MN definitions (post KK group all ‘underweight for age’) • Small sample size • Enzymes improved after acute recovery, but not to control levels • Enzymes correlated with baseline plasma albumin more than patient groups
Bartels *et al.*, 2017^(16)^	RCT -single blind	Malawi High	6–60 months	90 MN	Inclusion: hospitalised with SAM Exclusion: malaria or severe infections	• SAM: WHO criteria^(91)^	• Admission, d14, d28 • Faecal elastase (FE) • Exocrine pancreatic insufficiency (EPI): EPI: FE < 200 μg/g Severe EPI: FE < 100 μg/g	Pancreatic enzyme replacement therapy *v.* standard care	• At admission, EPI = 83·1 %; severe EPI = 69 % and median FE lower in oedematous *v.* non-oedematous MN • After treatment, FE increased in all children unrelated to enzyme therapy and more in those with non-oedematous MN • Most with oedema at baseline remained with EPI or severe EPI at d28.	• RCT 1º outcome of weight gain not affected but ↓ time to discharge and lower mortality in enzyme therapy group • Nutritional status of children after intervention not clear. • No modifying effects of HIV or diarrhoea
Bartels *et al.*, 2016^(17)^	Clinical cohort	Malawi Low	IQR 16–27 months	89 MN, incl 56 with oedema	Inclusion: 6–60 months, hospital with SAM +/– HIV Exclusion: previous admission within 1 years, severe neurological or haemodynamic illness	• SAM: WHO criteria^(91)^	• Admission and 3 d post-stabilisation • Faecal elastase (FE) • EPI: FE < 200 μg/g • Severe EPI: FE < 100 μg/g • Serum trypsinogen and amylase	NR carried out according to WHO^(1)^	• At admission, EPI = 92·2 %; severe EPI = 76·6 %. • Severe EPI more common in oedematous than non-oedematous MN (88 % *v.* 59 %); • d3 *v.* d0, FE modestly ↑ in all children but 83 % still EPI at d3; • Higher trypsinogen but not amylase in oedematous MN; • No association between trypsinogen and FE.	• No modifying effects of age, sex and HIV
Briars *et al.*, 1998^(18)^	Cross-sectional	Australia Low	0–3 years and 6 months –15 years	Two groups in two sites • 472 0–3 years; • 187 0·5–< 16 years;	Aboriginal children admitted to hospital	• Severe MN: WAZ < –2·0 • Mod MN: WAZ − 1·0 to −2·0 • Control: WAZ > –1·0	• Hospital admission • Whole-blood trypsinogen	None	• No difference in trypsinogen concentration between MN groups; • inverse correlation between trypsinogen and WAZ; • Higher trypsinogen in admissions associated with gastroenteritis	• Non-standard categories of MN; • Not all statistics reported
Danus *et al.*, 1970^(19)^	Clinical cohort	Chile V. Low	< 2 years	• 10 MN, • 7 non-MN hospital controls	Not specified	• Marasmic: grade 3 MN • Well nourished not defined.	• Admission, d4, d30 of NR • Pancreatic secretion volume, duodenal bicarbonate, amylase, lipase before and after SST (stimulation with secretin, cholecystokinin)	NR but no details given	• Comparable pancreas secretion volume in MN and controls before and after SST • bicarbonate increased 5–6-fold after stimulation in all children; • Amylase & lipase, basal and after SST ↓ in MN at admission and after NR compared with controls	• unclear definitions of nutritional status • Formal statistics not reported.
Durie *et al.*, 1985^(20)^	Clinical cohort	Canada V. Low	0·1–3·8 years (MN) 0·1–5 years healthy controls	• 25 severe MN • 23 Moderate MN • 2 Mild-MN • 38 healthy controls	Inclusion: • Hospital with wasting Exclusion criteria unclear	• Severe MN: <= 75 % of ideal weight-for-height (WfH) • Moderate MN: 75–85 % of WfH • Mild-MN: 85–90 % of WfH	• Admission and after NR • Serum trypsinogen.	Oral or intravenous NR but no details given	• Trypsinogen positively associated with severity of MN and ↑ compared with controls at admission • After NR, trypsinogen tended to revert toward normal, especially in those with greatest nutritional improvement • Fat malabsorption documented in 17/43 MN	• Unclear definition of MN • Reasons for hospitalisation missing • Various primary clinical causes of MN • No details of healthy controls • No details of NR • No renal impairment which could ↑ trypsinogen
El-Hodhod *et al.*, 2005^(21)^	Clinical cohort	Egypt V. Low	Mean for MN 11·8 months, Control mean 14·8 months	• 33 MN: 10 KK, 15 MS,8 marasmic-KK • 12 age- & sex-matched healthy controls	Inclusion: hospital with MN Exclusion unclear	• Wellcome 1970^(92)^	• Admission & after 3–6 months NR • Pancreatic head size by ultrasonography; • Serum amylase and lipase	NR carried out according to the WHO	• ↓ serum amylase, lipase & pancreas head size in all MN groups compared with controls, before NR • Pancreas head size, amylase, lipase ↑ in all MN groups after NR, increasing with duration of treatment	• Multiple statistical tests with limited sample size • Controls assessed once only so age-associated changes not accounted for in post-NR comparisons
Keni *et al.*, 1995^(22)^	Cross-sectional	India V. Low	9 months–9 years	• 23 MN of varying severity inc 12 marasmic-KK • 5 non-MN controls	Inclusion: 9 months–9 years, MN hospitalised with recurrent diarrhoea; Exclusion: cystic fibrosis	• NCHS WfH • 4 MN grades	• Admission • Pancreatic water electrolytes and trypsin output (units/kg/h) during SST, assessed in duodenal fluid	Not reported	• No consistent effect on trypsinogen across MN grades. • water output increased in MN with bicarbonate relative to water output but decreased in severe MN associated with loss of K+ in severe MN	• Results may not be applicable to non-persistent diarrhoea cases • Multiple statistical tests with limited sample size
Mehta *et al.*,1984^(23)^	Non-randomised 3-arm trial in MN plus controls	India Low	9–42 months	56 MN 15 age-matched Not controls	Inclusion: Hospitalised with MS Exclusion: no major infection	• MN weight < 60 % of median Boston standards • Controls weight > 80 % of Boston median	• Admission and after 2 weeks of NR • Duodenal amylase, lipase and trypsin activity, duodenal pH after pancreatic stimulation (milk).	• High protein diet (*n* 16): • High-fat diet (*n* 20); • High carbohydrate diet (*n* 20): • Each diet also sub-divided into further different compositions	• ↓ pH, duodenal amylase, lipase and trypsin activity in MN compared with controls before NR; • High protein diet ↑ pH and all enzyme activities compared with baseline; • High-fat diet ↑ lipase activity only compared with baseline	• Selection of controls • Reports enzyme activities/dl not amounts • No comparisons with controls post NR • No report of degree of nutritional recovery
Sauniere *et al.*, 1986^(24)^	Cross-sectional	France & Ivory Coast Low	1–8 years	• 15 Ivorian MN (KK) • 10 recovered KK; • 73 hospital controls (62 French, 11 Ivorian)	Inclusion • Current KK • recovered KK; • recurrent KK; • Non-MN control from acute conditions or trauma Exclusion criteria not specified.	KK by clinical criteria	• Current MN at admission or after recovery from KK • Pancreatic amylase, lipase, phospholipase trypsin, chymotrypsin electrolytes in duodenal fluid volume after SST	Not reported	• ↓ Lipase, trypsin and phospholipase in MN compared with controls • recovered MN not different from controls • ↓bicarbonate, Cl-, lipase, phospholipase in African control *v.* European control	• Europeans and Africans differed non-consistently across tests attributed to diet not ethnicity • Statistical tests unclear • Small sample size for MN, especially for recurrent MN
Sauniere *et al.*, 1988^(25)^	RCT in MN plus controls	Senegal, Ivory Coast, France Low	1–3 years	• 28 MN (KK) • 21 African controls • 31 French controls	• Specific inclusion and exclusion criteria not described • Controls mix of relatives of MN, hospital controls with no medical or surgical disease, or French	KK by clinical criteria	• Admission and after 5 d (Ivory Coast) or 28 d (Senegal) of NR • Pancreatic secretion volume, duodenal pH, electrolytes, amylase, chymotrypsin, lipase, phospholipase and trypsin after SST	• NR locally sourced diet • Random assignment to pancreatic enzyme replacement therapy (porcine pancreas powder)	• Secretion of enzymes and electrolytes ↓ in African controls (esp Senegalese) than French controls (inc when adjusted for body weight) • In Ivory Coast MN had ↓pancreas enzyme secretion than controls which significantly improved after 5 d of NR but not to French or African control levels. • In Senegal MN, severe pancreas enzyme deficiency not improved by 28 d NR • No effects of pancreatic enzyme replacement therapy	• Different NR in two countries with very different outcomes. • Pancreatic assessments standardised across sites • Multiple testing across many small groups • Low pancreas enzymes in African controls compared with French suggest other environmental factors than MN are key
Thompson *et al.*, 1952^(26)^	Non-randomised multiple arm trial	Uganda V. Low	9–58 months	59 MN (KK), 24 non-MN hospital control	Inclusion: pitting oedema, hair changes, low weight Controls: normal hair, no oedema, hospitalised for other conditions Exclusion criteria not specified	• KK by clinical diagnosis	• At admission to hospital (1–5 d) and discharge after NR (7–51 d) (*n* 40) • Duodenal amylase and lipase	• NR: hospital diet with comparisons of groups assigned to extra milk or other protein supplements	• At admission, ↓ amylase and lipase in KK *v.* control • At discharge, lipase ↑ similar to controls and amylase ↑ > controls • No differences in change of amylase/lipase between NR interventions.	• Statistical analysis details missing • Little information about the non-MN controls, e.g. weight not given
Widodo *et al.*, 2016^(27)^	Cross-sectional	Indonesia Low	6–60 months	• 31 MN • 120 non-MN apparently healthy hospital controls	Inclusion: 6–60 months at 2 hospitals in- and outpatient clinics Exclusion: • EPI at birth • Inflammatory bowel disease or other chronic diarrhoea • recent medications	(moderate or severe MN not defined	• Faecal elastase (FE)	None	• No difference in FE in MN *v.* controls	• Study also recruited children with persistent diarrhoea – data not included here • Group with persistent diarrhoea and MN had lowest FE
Endocrine pancreas function										
Adegbenro *et al.*, 1991^(29)^	Cross-sectional	Nigeria/Low	1–5 years	25 KK, 25 MS, 25 non-MN healthy controls	Inclusion: not specified Controls same age group, well nourished, clinically stable; Exclusion: Marasmic-KK	• MN by Wellcome Trust^(92)^	• FBG and HbA1c	None	• ↑ HbA1c in KK *v.* controls but comparable in MS *v.* controls • ↓ FBG in all MN *v.* controls; • In MN, HbA1c inversely associated with to FBG	• Relatively small sample size, in relation to observed variability in FBG and HbA1c. • Only 1 KK had hypoglycaemia • MN children did not require hospitalization
Becker *et al.*, 1971^(30)^	Clinical cohort acutely and long-term follow-up	South Africa Low	8–38 months acutely; 7–12 years at follow-up	Acute MN • 38 KK • 16 MS • 10 age-matched non-MN controls Follow-up • 10 recovered MN • 10 closest siblings as controls	Inclusion: MS or KK Controls with wt > 3rd Boston percentile Exclusion: obvious infection, anaemia, or gross diarrhoea Follow-up: Hospitalised for KK 10 years earlier	• KK defined clinically MS: <= 60 % WfA without oedema	• Admission, recovery (3–6 weeks of NR), 2–10 months of NR (in persistent abnormal) • Ratio of insulin AUC : glucose AUC) in OGTT and/or IVGTT • OGTT and glucagon in subset • 10 years post KK Ratio of Insulin AUC:glucose AUC in 90 min IVGTT	• Locally prepared NR	• Insulin and insulin/glucose AUC in OGTT very low in MN on admission and ↑after 3–6 weeks • Insulin and insulin/glucose AUC in IVGTT less affected than OGTT in MN and ↑ after 3–6 weeks NR • KK ↑ more for Insulin:glucose AUC than MS • 16/49 at 3–6 weeks recovery Insulin abnormal • Some low insulin responses still evident after 2–10 months NR but similar to controls • At long-term follow-up, no difference between recovered MN and control in insulin/glucose AUC	• abnormal Insulin < 20 uU/ml not based on controls – noted 2/10 controls probably not normal; • at follow-up 6/10 MN still had low WfA • normal IVGTT but low insulin in OGTT suggests enterocyte rather than pancreas problem
Becker *et al.*, 1972^(31)^	Clinical cohort	South Africa V. Low	8–38 months	• 38 KK • 16 MS • 10 age-matched non-MN controls	Inclusion: MS or KK Controls with wt > 3rd Boston percentile Exclusion: obvious infection, anaemia, or gross diarrhoea	• KK defined clinically • MS: <= 60 % WfA without oedema	• Admission and after 1–6 months NR • Glucose & insulin in OGTT and/or IVGTT	• Locally prepared NR	• Analysed as patterns of insulin responses to OGTT/IVGTT; 4 patterns: (a) most common flat low insulin curve after OGTT and flat/normal after IVGTT (*n* 35/54 MN); (b) sustained insulin curve after OGTT & IVGTT; (c) delayed insulin response after OGTT & normal IVGTT; (d) delayed insulin response after IVGTT only – in 7 with MS-KK (denominator not reported) • After NR, all patterns were normal	• Note overlap of pops with[29] • Normal response defined from a typical normal control • Pattern classifications based on OGTT and IVGTT, but only 14 children had both tests. • No statistics and unclear denominators
Becker *et al.*, 1975^(40)^	RCT–2 arm	South Africa Low	6–27 months	10 MN, no controls	Inclusion: Hospitalised children with KK • Exclusion: overt infection • severe gastroenteritis	• KK defined clinically	• Admission, d2, d5 and discharge after 3–5 weeks NR • Insulin:glucose AUC ratio (IGR) and insulin AUC during OGTT (60 min)	• Alternatively assigned to high oral potassium (*n* 5) or conventional potassium supplementation (*n* 5)	• Early insulin release (AUC in 60 min) during OGTT low in MN • Insulin AUC & IGR↑ by 3–5 weeks NR but no controls so unclear if it reached normal levels • Insulin AUC 60 min ↑ in high *v.* low K supp group	• Small sample size • Not randomised
Becker *et al.*, 1975^(41)^	Clinical cohort	South Africa V. Low	8–38 months	7 MS, 28 KK or MS-KK	Inclusion: MS or KK Exclusion: obvious infection, severe anaemia, gross diarrhoea	• KK defined clinically • MS: ≤ 60 % WfA without oedema	• At admission, subsets repeated at 24 or 72 hr after either albumin, amino acid infusion or milk feed and at clinical recovery (3–6 w) • Insulin insulin:glucose AUC (IGR) and glucose disappearance rate during IVGTT (120 min)	Standard local hospital diet	• Glucose disappearance rate low at admission but increased with NR • Peak insulin associated with glucose clearance rate only at admission • Peak insulin and albumin correlated at admission but no consistent effects of acute infusions, except possible ↑ in IGR after aa infusion	• Note sub-pop of [29] Multiple testing • No controls • Main purpose of analysis is to assess correlations of insulin and glucose responses to markers of protein or aa status
Bowie *et al.*, 1964^(32)^	Cross-sectional	USA V. Low	Infants and children (ages not given)	• 24 KK • 10 MS • 5 normal weight controls	Inclusion and exclusion criteria unclear	• KK defined clinically • MS: low weight and no oedema	• After 3 d admission and milk feeding • Glucose disappearance rate during IVGTT • 4 children also given insulin infusion	Milk feeding	• Slower glucose disappearance in KK than in MS or well-nourished • Slow disappearance rate in KK not improved by insulin infusion	• Incomplete study methods • No statistical analysis • factors other than insulin production may be responsible for poor glucose utilisation in KK
Fransis-Emmanuel *et al.*, 2014^(44)^	Case-control	Jamaica Medium	6–18 months	• 38 KK • 42 MS • 70 community control • 40 birth weight-matched control	Inclusion: • Hospitalised 6–18 months children with KK and MS during 1963–1992 • Controls: Never experienced MN Exclusion: • Acutely ill, pregnant, lactating, on glucocorticoid, or had haemoglobinopathy	• KK & MS by welcome criteria	• FBG and fasting insulin • Glucose and insulin during OGTT	Not applicable	• No group differences in FBG • ↑ peak levels of glucose during OGTT in post-MS *v.* post-KK • ↓ Insulin sensitivity in post-MS *v.* post-KK • ↓ Insulinogenic and oral disposition index in post-MS *v.* all	• Findings are unlikely confounded by survival effect
Garg *et al.*, 1989^(35)^	Cross-sectional	India Low	2–10 years	• 15 MN, 5 at each of grades II, III, IV • 5 normal controls	Inclusion: • Children with varying grades MN • Adequately nourished controls Exclusion: No other illnesses	• MN defined by % of reference weight • Normal >= 80 % • Grade II 60–69 % • Grade III: 50–59 % • Grade IV: < 50 %	• FBG and fasting insulin • Glucose and insulin during OGTT	Not applicable	• No group differences in FBG and fasting insulin • ↑ Peak levels of glucose during OGTT in grades III and IV MN *v.* controls • ↑ Peak insulin during OGTT in grades III and IV MN *v.* controls	• Unclear from where and how the children were recruited • Unclear duration of MN or when pancreas assessments done • Authors said insulin responses were blunted in MN in relation to glucose levels but provided no analysis to this
Gonzalez-Barranco *et al.*, 2003^(45)^	Case–control	Mexico Medium	Mean age 4·5 (sd 3·1) months	• 52 MN • 50 healthy controls	Inclusion: Nondiabetic men with history of MN during 1st year of life Exclusion criteria not specified	• MN defined by % of reference weight • Degree I 76–90 % • Degree II 61–75 % • Degree III: 30–60 %	• FBG and insulin • AUC of glucose and insulin • Insulin sensitivity	• Not applicable	• ↑ Fasting and during OGTT glucose and insulin in post-MN *v.* controls • ↑ AUCO glucose and insulin in post-MN *v.* controls • ↓insulin sensitivity in post-MN *v.* controls	• Nondiabetic cases included • Cases were from lower socio-economic strata
Hadden *et al.*, 1967^(33)^	Clinical cohort	Uganda V. Low	Mean age 15 (sd 6) months	• 24 KK • 9 MS	Inclusion: ‘clinical and biochemical criteria’ for KK and MS but not described Exclusion criteria not specified	• KK defined clinically • MS: ↓ weight, no oedema	• Admission (d1) and several times up to d14 of NR • FBG and insulin • IVGTT at d1 and d14 • Glucose disappearance rate	• Locally sourced diet plus multivitamin supplements	• Limited differences in FBG between groups or over time • At d1 & d14 fasting insulin higher in KK than MS; • Insulin tended to normalise during NR • At admission, slow glucose disappearance in KK but improved after NR; glucose disappearance rate negatively correlated with fasting blood fatty acid level • Normal glucose disappearance in MS and no correlation with fatty acids	• Lacking statistical methods • No well-nourished controls; children own controls over time • Values at d14 judged to be comparable to normal • Association of glucose disappearance with fatty acids suggests insulin resistance related to high blood fatty acids in KK from metabolic block in utilisation
James *et al.*, 1970^(34)^	Cross-sectional	Jamaica V. Low	6–18 months	• 26 MN, • 28 treated MN • 5 non-MN hospital controls	Inclusion • Clinical MN diagnosis • Control: hospitalised for bronchitis with no clinical evidence of malnutrition. Exclusion criteria: infection, anaemia, severe diarrhoea or jaundice	• MN: mean 72 % expected WfH • Treated MN: 89 % expected WfH • Control 92 % expected WfH	• 3–18 d or 6–20 d after hospital admission • FBG • Insulin and glucose during IVGTT • Glucose disappearance rate • OGTT in subset of MN and treated children	• Locally sourced protein and energy supplement	• No group differences in FBG • Glucose disappearance low in untreated MN; increased with NR but not to control levels • Insulin during IVGTT low in untreated MN and increased slightly but not to control levels after NR	• Unclear definition of MN • Impaired glucose tolerance persisted after treatment, possibly due to persistent low insulin production
Milner *et al.*, 1971^(47)^	Clinical cohort	Jamaica V. Low	6–27 months	• 11 MS • 5 KK • 10 MS-KK	Inclusion: • MS: weight < 66 % expected, no oedema • KK: weight > 60 and expected, oedema • Marasmic KK: weight < 60 % expected, oedema	• MS, KK and marasmic KK based on % expected weight and oedema	• 1–2 d post-hospital admission and 6–12 weeks NR • FBG in all • Repeated IVGTT in 10 children • Repeated Glucagon injection in 9 children	• Local milk diet refeeding	• FBG similar at admission and post-NR • Fasting insulin higher after NR than at admission • Glucose tolerance improved post-NR • Insulin higher in IVGTT post-NR • Blood glucose after glucagon injection higher post-NR; no difference in insulin	• No healthy controls; children post-NR were their own controls • Multiple *t* tests for several analytes at different time points within IVGTT
Pereyra *et al.*, 2021^(46)^	Retrospective cohort	Chile Low	12 months	• Cohort-1:1232 participants born between 1974 & 1978; • Cohort-2:1000 participants born between 1988 and 1992	Inclusion: •Cohort-1: participants born between 1974 & 1978; •Cohort-2: participants born between 1988 and 1992 Exclusion • Missing data for birth weight, FPG and fasting insulin	• Stunted: LAZ < –2 sd; • Severe stunted: LAZ < –3 sd; • Underweight: WAZ < –2 sd; • Severe underweight: WAZ < –3 sd; • Wasted: WLZ < –2 sd; • Risk of wasting: WLZ < –1 sd; • Birth weight • Conditional growth: regressing weight/length on birth weight and earlier measure of weight and length	• At age 22–28 years • 12-h FPG; • Fasting insulin; • Homeostasis model assessment of insulin resistance (HOMA-IR); • Single point insulin sensitivity estimator (SPISE)	• N/A	• ↑ adulthood SPISE in underweight subjects; • Adulthood glycaemia positively associated wasting and at risk of wasting condition; • ↓Glycaemia adulthood associated with ↑ WLZ at 12 months	• Participants selected randomly; • Possibilities of selection bias due to missing data; 1070 inc in final analyses • Probability of error on routine records at birth and 12 months
Prinsloo *et al.*, 1971^(37)^	Clinical cohort	South Africa V. Low	Mean age 55·6 (sd 20·5) months	16 KK; 15 pellagra controls	Inclusion: • Hospitalised for KK • Control with pellagrous skin lesions without oedema Exclusion: • Patients moribund on admission	• KK defined clinically	• 2–5 d after admission and after 4–5 weeks NR • FBG • Glucose and insulin during IVGTT; and glucose disappearance rate	• Usual hospital diet plus milk, potassium, multivitamins	• At admission, no group difference in FBG but lower insulin and slower glucose disappearance in MN • After NR increased insulin and improved glucose disappearance, now similar to controls	• Authors stated pellagra controls had normal glucose tolerance • NR normalised insulin and glucose
Robinson *et al.*, 1982^(38)^	Non randomised 2-arm trial	Jamaica V. Low	6–20 months; median 12 months	20 MN 11 healthy adults to provide normal glucagon levels	Inclusion: Severe MN Exclusion criteria not specified	• Severe MN: expected WfH 52·6–83·6 %	• Admission and weekly intervals until recovery • FBG • Fasting glucagon and insulin	• Maintenance diet followed by 1 of 2 different locally made recovery diets (high carb *v..* high fat)	• FBG similar during MN and recovery • Low fasting glucagon, insulin during MN which increased during first few weeks of recovery but then declined slightly, correlated with growth rates and insulin: glucagon ratio increased at period of max growth rate	• Missing information about study participants and statistical methods • Adult controls for glucagon level; children also served as their own controls • No diff of diets on rate weight gain but high carbohydrate diet ↓ glucagon responses
Slone 1961^(36)^	Cross-sectional	South Africa V. Low	0·5–3 years	• 20 KK • 20 control	Inclusion: • KK Exclusion: dehydration	• KK by clinical diagnosis	• 2 d post-hospital admission • FBG in all • IVGTT in 9 MN	NA	• Low FBG in MN • Mild glucose intolerance in IVGTT but controls not studied	• No statistical analyses and no controls in IVGTT
Spoelstra *et al.*, 2012^(39)^	Cross-sectional	Malawi Medium	Mean age 25·3 (sd 15) months	• 6 KK/MS-KK • 8 MS • 3 non-MN hospital controls	Inclusion: • Children clinical diagnosed KK or MS. With or without HIV • Control: Minor orthopedic problem or recovered from respiratory illness Exclusion: • Multiple indicators of severe illness	• KK defined clinically • MS: WAZ < –3 or MUAC < 11 cm	• During stabilisation phase of NR • Glucose infusion (4 h) and OGTT (at 2 h of glucose infusion) with doubly isotopically labelled glucose • Modelled glucose clearance in OGTT	WHO recommended NR ^(1)^	• Oral rate of glucose appearance reduced in KK; • Lower glucose clearance in both MN groups with low insulin response. Low ratio of glucose clearance AUC: insulin AUC –strongly suggesting B-cell impairment not insulin resistance • Correlation between plasma albumin and glucose clearance rate	• Glucose clearance rate excludes endogenously produced glucose • investigation using isotopically labelled • Both HIV-infected and uninfected children recruited • Inflammation possibly associated with lower albumin, or oxidative stress could have contributed to low glucose clearance
Cook *et al.*, 1967^(42)^	Cross-sectional	Uganda V. Low	• Ages not given, likely 8–16 years since MN aged 1–4·4 years when MN	• 14 recovered KK, 20 community control	Inclusion: • History of hospitalisation for KK • Community control with no history of KK Exclusion criteria not specified.	• KK by clinical criteria	• 6–12 years post hospitalisation for KK • Glucose disposal rate during IVGTT	NA	• Slower glucose disposal rate in recovered KK *v.* controls	• Some study details missing
Kajubi *et al.*, 1972^(43)^	Cross-sectional	Uganda Low	11–19 years after hospitalised with MN aged 1·5–3 years	• 15 post-KK • 8 controls	Inclusion: • Previously registered as KK • Exclusion criteria not specified Controls – convenience, no reported history of KK	• KK by clinical criteria	• Age 11–19 years • OGTT for 40 min with glucagon and tolbutamide given after 30 min to elicit maximum insulin release	NA	• ↓ Fasting insulin in post- KK *v.* controls • ↑ Glucose during OGTT not significantly different but did not calculate AUC or glucose disappearance rate • Comparable maximum insulin in recovered KK and control	• Suggest the comparable maximum insulin indicates no permanent damage to the pancreas • Statistical analysis methods missing. • No differences in weight, height and Hb between post-KK and control group

FU, follow-up; MN, malnutrition or malnourished; MS, marasmus; KK, kwashiorkor; SST, stimulation test with secretin or cholecystokinin; SAM, severe acute malnutrition; RCT, randomised controlled trial; WAZ; weight-for-age Z score; FBG, fasting blood or plasma glucose; FE, faecal elastase-1; WfA, weight-for-age using percentiles; WfH, weight-for-height/length using percentiles; HbA1c, glycosylated Hb; IVGTT, intravenous glucose tolerance test; LAZ, length-for-age; MUAC, mid-upper arm circumference; OGTT; oral glucose tolerance test; .

**Table 2.** Association between late childhood or adult malnutrition and pancreatic function

Author and reference	Study design	Country/quality assessment	Age when MN	Number of study participants	Inclusion/exclusion criteria	Definitions of MN	Pancreatic Function times and tests	Intervention/treatments	Main findings	Comments
Exocrine pancreas function										
Martinez-Olmos *et al.*, 2013^(50)^	Clinical cohort	Spain V. Low	26 years	• 10 AN	Inclusion: • Hospitalised for AN • BMI < 17·1 kg/m^2^ Exclusion: Other chronic disease	• Hospitalised for AN	• Admission and at discharge when BMI > 20 kg/m^2^ • Faecal elastase • Also measured triglyceride and D-xylose absorption to study gut function	Therapy for AN	• No difference in elastase before and after weight gain	• Participants after recovery were their own controls • Triglyceride and D-xylose tests were normal
Tandon *et al.*, 1969^(48)^	Cross-sectional	India V. Low	22–55 years	• 16 MN • 10 normal weight hospital controls	Inclusion: • MN in hospital • Control: ambulatory outpatients without symptoms or signs of disease; Exclusion • If pancreatic or biliary disease	• Muscle wasting or peripheral oedema defined clinically • Low serum albumin	• Duodenal juice amylase, lipase, trypsin after SST.	NA	• ↓ Trypsin and lipase, concentration both basal and stimulated in MN *v.* control • Amylase lower in MN after stimulation but not in basal samples • Steatorrhoea in MN patients	• Conducted when serum albumin considered to indicate protein deficiency • Authors suggested larger decreases in stimulated than basal enzymes indicated low pancreatic reserve
Tandon *et al.*, 1970^(49)^	Clinical cohort	India V. Low	22–55 years	• 8 MN • 10 healthy controls	Inclusion • Patients clinically diagnosed Exclusion • clinical history of alcoholism or chronic pancreas or biliary tract disorders	• Pallor, dry scaly skin, muscular wasting and generalised oedema.	• Admission and after 12–14 weeks treatment • Duodenal juice lipase, trypsin, amylase, basal and post SST	Local protein-energy rich diet plus iron and vitamins	• ↓Trypsin at admission, both basal and stimulated; little increase with NR • Lipase low before NR but normalised after NR • Amylase low post-stimulation at admission; normalised after NR	• Non-standard definition of MN; dietary history suggested prolonged low intakes • Multiple *t* tests with small numbers of participants
Endocrine pancreas function										
Blickle *et al.*, 1984^(51)^	Cross-sectional	France V. Low	19–25 years	• 26 AN • 14 normal weight controls	• Women in hospital with AN • Controls in hospital for minor disorders	• Clinical AN, 44–82 % ideal weight	• Within a few days of hospital admission • Glucose, insulin and glucagon fasting and during IVGTT and arginine perfusion • Insulin/glucose ratio	Not applicable	• Fasting glucose and insulin correlated with % ideal weight • Low glucose in IVGTT in AN *v..* control but normal glucose disappearance rate • ↓insulin and insulin/glucose ratio during IVGTT and arginine infusion in AN • No difference in fasting glucagon or glucagon response in IVGTT in AN *v.* control	• Wide variability of plasma glucagon may have obscured group differences • Also measured growth hormone during the tests
Brown *et al.*, 2003^(57)^	Cross-sectional	UK Low	29·4 (sd 8·2) years	• 18 recovered AN • 18 healthy controls	Inclusion • women recovered from AN with BMI > 18·5 kg/m^2^, resumption of menses and normal eating habits • Controls: no history of eating disorder; similar BMI to recovered AN Exclusion Bulimic patients	• Clinical AN	• Unclear time post AN diagnosis or recovery • Glucose and insulin fasting and after a standard meal • Glucose/insulin ratio	Not applicable	• Comparable FBG and glucose response to meal; • ↓Fasting insulin and slow rise in insulin in response to meal in recovered AN group • ↑Fasting glucose/insulin ratio in recovered AN group, suggesting ↑ insulin sensitivity in face of ↓insulin production	• Missing details of AN severity and time since recovery from AN • Also analysed leptin and *β*-hydroxybutyrate
Casper *et al.*, 1988^(58)^	Cross-sectional from prospective cohort	USA Medium	8–10 years before curreent age of 21–40 years	• 19 AN recovered • 7 AN unrecovered • 14 age-matched controls	Inclusion: • Women recovered from AN with normal body weight, resumption of menses • Women unrecovered from AN with weight < 85 % expected, sporadic or absent menses • Controls: women with normal body weight Exclusion: • taking medication	• Clinical AN	• 8–10 years after AN diagnosis • Fasting plasma glucose and insulin • OGTT • IGT: FPG < 140 mg/dl and glucose 140–200 mg/dl during OGTT • DM: FPG > 140 mg/dl or glucose > 200 mg/dl during OGTT •	Given standard diet for 5 d before testing	• Comparable FPG and fasting insulin in all groups • ↑ maximum glucose during OGTT in unrecovered AN but not recovered AN • Slow rise insulin during OGTT in unrecovered AN but not in recovered AN *v.* control • DM plus IGT diagnosis greater in unrecovered and recovered AN than control	• Few long-term glucose metabolism problems in recovered AN • Authors also investigated glucose associations with psychological variables and levels of cortisol, growth hormone and fatty acids
Fujii *et al.*, 1989^(55)^	Clinical cohort	Japan Medium	13–35 years	• 16 females with AN • 8 age-matched normal weight females	Inclusion: • Women with AN • Control: healthy well-nourished, without family history of diabetes	• Clinically diagnosed AN	• Within a week of admission and after 5 months • Plasma glucose and glucagon in response to insulin infusion • Plasma glucose, glucagon and insulin in response to arginine infusion (pre-treatment only)	5 months treatment for AN	• Lower FPG and delayed glucose recovery after insulin in AN *v.* control; returned to normal after NR • Low fasting glucagon and glucagon response to insulin-induced hypoglycaemia in AN *v.* control; normalised after NR • After arginine infusion, no difference in glucose or glucagon response but insulin response lower in AN *v.* controls	• Focus on glucagon production but insulin production also low in AN
Filteau *et al.*, 2021^(65)^	Prospective cohort	Tanzania Medium	> 18 years	• 630	Inclusion • Participated in previous research projects on malnutrition Exclusion • Missing information on glucose and insulin	• SMN: BMI < 17 kg/m^2^; • MN: BMI =≥ 17–< 18·5 kg/m^2^; • Normal weight: BMI ≥ 18·5 kg/m^2^	• HbA1c, glucose and plasma insulin fasting and during OGTT	Not applicable	• Prior MN associated with lower insulin concentration in men only • Current MN had lower insulin concentration irrespective of sex	• Duration of being MN unclear
Kanis *et al.*, 1974^(64)^	Clinical cohort	UK V. Low	15–43 years	• 24 AN, but OGTT data shown only for 13 patients followedup	Inclusion: • hospitalised with AN • female Exclusion: • Male AN patients	• Clinical AN	• At admission & follow-up after 5–15 months • Glucose and insulin fasting and during OGTT	Psychiatric treatment for AN	• Blood glucose remained high longer during OGTT before treatment than after • Plasma insulin remained high longer before than after treatment	• No healthy controls • Glucose and insulin results shown only for subset of AN patients
Kobayashi *et al.*, 1992^(56)^	Clinical cohort	Japan V. Low	21 years	• 14 AN • 6 bulimic • 6 age-matched healthy controls	Inclusion: • hospitalised with AN or bulimia • Control without personal and family history of diabetes Exclusion: • Liver disease	• Clinical AN or bulimia	• Within 2 weeks of hospitalisation and after weight gain (eight AN patients only) • Blood glucose, serum insulin and C-peptide fasting and after IV glucagon	Local dietary therapy for AN	• Comparable glucose fasting and after glucagon in AN, bulimia and control • Lower insulin and C-peptide after i.v. glucagon in AN *v.* controls • No change in glucose, insulin or C-peptide in AN after weight gain.	• No description of study population
Kumai *et al.*, 1988^(52)^	Clinical cohort	Japan Low	22 years	• 25 AN patients • 15 age-matched healthy controls	Inclusion: • Female patients diagnosed with AN • Healthy controls within 10 % of ideal body weight without family history of DM Exclusion: • males • taking medication	• Clinically diagnosed AN	• Within 7 d of admission and before discharge, approximately 6 months later, when weight similar to controls • Glucose, insulin and glucagon fasting and during OGTT	NR and other therapy for AN	• Lower FPG but raised glucose during OGTT at admission; both became more normal after NR • Low fasting insulin & delayed and lower response (levels and total AUC) during OGTT; still abnormal after NR • Glucagon low fasting and increased, not decreased, during OGTT at admission; normalised post NR	• Large decreases in insulin not improved by NR although glucose did improve after NR suggesting residual low insulin production; • possibly ↑ peripheral insulin sensitivity post NR
Ji *et al.*, 2016^(59)^	Population-based cohort	Sweden High	Median age 17 years for females, 9 years for males	• 17 135 AN • 12 910 sibling pairs without AN as controls	Inclusion: • Prior hospitalisation for AN based on national register Exclusion: • 12 participants with prior DM	• Hospitalised with clinical AN 1964–2010; diagnosis based on ICD codes	• DM incidence on national register; post age 39 years to exclude type 1 DM		• During 259 088 p-y follow-up, 34 individuals developed DM • 30 % ↓risk of DM in AN *v.* general population • Comparable risk of DM in AN *v.* siblings	• Investigating whether caloric restriction decreases chronic disease risk • Low DM incidence, possibly related to low prevalence of obesity in former AN patients
Letiexhe *et al.*, 1997^(60)^	Cross-sectional	Belgium Low	16–39 years	• 9 AN • 9 age-matched healthy controls	Inclusion: • Female patients with AN with normal renal and liver function • Female healthy controls Exclusion: • Bulimia nervosa • Physical exercise 2 days before test • Medications that interfere glucose metabolism	• Clinical AN, BMI 10·2–15·7 kg/m^2^	• At hospital admission, before NR • Glucose, insulin and C-peptide fasting and during IVGTT, AUCs calculated • Insulin sensitivity index • Glucose effectiveness index • Insulin metabolic clearance rate • Glucose metabolic clearance rate		• Comparable fasting levels and AUC for glucose and C-peptide in both groups • ↓ Fasting insulin and insulin AUC in AN *v.* control • ↑ Insulin metabolic clearance rate • ↓ Glucose tolerance in AN *v.* control	• Multiple indices of insulin secretion and clearance • Suggested the low insulin in AN is not due to increased insulin clearance, but to low insulin secretion • Wide range of duration of AN
Sizonenko *et al.*, 1975^(54)^	Cross-sectional	Switzerland V. Low	AN 14 years	• 6 AN • 10 normal controls • 32 patients with endocrine abnormalities not relevant here	Girls hospitalised for AN	• Clinical AN, < 65 % ideal weight and amenorrhoea	• Unclear time of tests with respect to hospital admission • Glucose, insulin and glucagon fasting and during arginine infusion	Not applicable	• Lower FBG and glucose during arginine infusion in AN *v.* control • Lower insulin fasting and during arginine infusion in AN *v.* control • Comparable fasting glucagon in AN *v.* control; glucagon remained high longer during infusion in AN than controls but not significantly so	• Statistical analyses unclear • Study underpowered for AN *v.* control • Focus of study was hormonal growth problems
Wallensteen *et al.*, 1991^(53)^	Cross-sectional	Sweden Low	AN 13–16 years	• 7 AN • 32 obese children • 16 healthy controls	Inclusion • AN from inpatient clinics • Obese from outpatient clinics • Healthy controls studied previously in same laboratory • No clear exclusion criteria	• Clinical AN	• AN patients when stable weight during hospitalisation • 24 h urinary C-peptide excretion	Not applicable	• Comparable total urinary C-peptide in AN *v.* control but increased in AN when calculated per kg weight	• Unclear at what stage of hospitalisation AN patients studied • Obese children were main focus of the study
Zuniga-Guajardo *et al.*, 1986^(61)^	Clinical cohort	Canada V. Low	25·2 (sd, 1·9) years	• 9 AN, including bulimia • 7 healthy controls	Inclusion: • Female patients diagnosed with AN or bulimia • Female healthy controls Exclusion: • Taking medications • associated illnesses	• Clinical AN and bulimia	• At outpatient visit; four patients studied again post-treatment • Fasting glucose, insulin, C-peptide • Glucose and insulin infusion for euglycaemic clamp • Metabolic clearance rate of glucose and insulin • M/I ratio: glucose metabolised/ unit insulin	Therapy for AN	• Lower FPG, fasting insulin and C-peptide in AN patient *v.* control • Higher glucose metabolic clearance rate in AN *v.* control • Higher insulin sensitivity in AN *v.* control, based on euglycaemic clamp test • After treatment, FPG, insulin and C-peptide concentration normalised in AN	• Unclear how participants selected initially and after treatment;
Sathiaraj *et al.*, 2010^(62)^	case-control	India medium	32·1 (sd 14) years	• 89 tropical pancreatitis of < 1 year duration • 101 age- and sex-matched community healthy controls	Inclusion • Tropical pancreatitis, including calcification or abnormalities on ultrasound, CT scan or endoscopy • Duration < 1-year; Exclusion • Acute exacerbation of pancreatitis, alcoholic liver disease, renal failure, pancreatic cancer, tuberculosis, HIV/AIDS, pregnancy	• MN: BMI < 18·5 kg/m^2^; • Normal weight: BMI = 18·5–24·9 kg/m^2^; • Overweight and obese: BMI > 25 kg/m^2^	• At admission • DM: FBG > 126 mg/dl and 2-h postprandial glucose > 200 mg/dl	Not applicable	• No difference in % MN pre-pancreatitis compared with controls. • Generally, weight loss occurred in pancreatitis as a result of low diet intake after disease onset, i.e. MN a consequence, not a cause of tropical pancreatitis • 13·5 % of tropical pancreatitis had DM	
Smith *et al.*, 1975^(63)^	Clinical cohort	India V. Low	Adults, age not specified	• 17 MN	• No clear inclusion or exclusion criteria; • MN patients from outpatient clinics, public places and refugee camps	MN definition not given but most had oedema.	• Admission and after 2–4 m of NR • FBG • Insulin and glucose during IVGTT and arginine infusion • Glucose disposal rate (% fall of blood glucose/min): during IVGTT.	Locally sourced high protein, high energy supplement	• Lower FBG before NR than after • ↓glucose disposal rate on admission *v.* after NR • At admission compared with post-NR, insulin during IVGTT had a slow rise but stayed high for longer • Both glucose and insulin responses to arginine blunted in MN compared with after NR	• No well-nourished comparison group; patients after NR were their own controls • Missing age and other participant details • Missing statistical methods

MN, malnutrition/malnourished; AN, anorexia nervosa; SMN, severe malnutrition; SST, secretin stimulation test; NR, nutrition rehabilitation; IVGTT; intravenous glucose tolerance test; DM, diabetes mellitus; FPG, fasting plasma glucose; IGT, impaired glucose tolerance; OGTT; oral glucose tolerance test; FBG, fasting blood glucose; ICD, International Classification of Diseases.

**Table 3.** Association between famine experience during childhood and adult endocrine pancreatic function

Author and reference	Study design	Country/quality assessment	Age when exposed to famine	Age at pancreas assessment	Number of study participants	Inclusion/exclusion criteria	Pancreatic function tests	Main findings	Comments
Finer *et al.*, 2016^(66)^	Cross-sectional study	Bangladesh Medium	• Postnatal (born 1–2 years before start of famine) • Fetal (exposed during gestation) • Unexposed (conceived 6 months to 2 years after famine) • Older children exposed when > 16 years	25–64 years	• 81 postnatal exposure • 40 fetal exposure • 70 unexposed • 112 exposed after age 16 years	Inclusion: • Famine exposure defined by date of birth with respect to famine in July 1974–June 1975	• OGTT (0 and 120 min glucose) and standard criteria of: • Impaired fasting glucose (IFG) 0 min glucose 5·6 – 6·9 mmol/l • Impaired glucose tolerance (IGT): 120 min glucose 7·8–11·0 mmol/l • DM: 120 min glucose ≥ 11·1 mmol/l	• More underweight in fetal exposed • More overweight in postnatal exposed • No overall differences in glucose outcomes but interaction with current BMI and 120 min blood glucose: underweight fetal exposed higher than other groups	• No differences in glucose outcomes between postnatal exposed and unexposed • Missing many statistical details and use of multiple post-hoc subgroup analyses • Likely survivor bias in those famine-exposed
Hult *et al.*, 2010^(67)^	Cross-sectional study	Nigeria High	• Early childhood (born 1965–1967) • Fetal and infant (born 1968–1970) • Unexposed born post famine transition period (1971–1973)	40–43 years	388 childhood exposure 292 fetal and infant exposure 486 unexposed	• Famine exposure defined by date of birth with respect to famine in 1967–70 • Participants born during transition period, February–December 1970, excluded.	• Random plasma glucose (RPG) • DM: RPG >= 11·1 mmol/l; • IGT: RPG 7·8–11·0 mmol/l	• Comparable risk for DM and IGT in childhood exposed and unexposed, but higher mean RPG and risk of IGT in fetal and infant famine exposure group compared with non-exposed • Similar OR seen for those with BMI < or > 25 kg/m^2^	• No association of RPG with famine exposure in childhood but exposure in fetal or infant life increased risk • Known survivor bias, i.e. famine-exposed may have died in infancy or childhood.
Li *et al.*, 2010^(68)^	Cross-sectional study. Subset of 2002 China National Nutrition & Health Survey	China High	• Childhood exposed born October 1952–September 1958, divided in 2 years age bands for exposure in late childhood, mid-childhood, early childhood • Fetal and infant born October 1959–September 1961 • Unexposed born post famine, October 1962–September 1964	38–49 years	• 1673 late childhood • 1588 mid-childhood • 1654 early childhood • 1005 fetal and infant • 1954 unexposed	• Famine exposure defined by date of birth with respect to famine in 1959–61 • Rural residence • Participants born during transition periods, October 1958–September 1959 and October 1961–September 1962 excluded	• FPG • OGTT in people with FPG > 5·5 mmol/l • DM: FPG > 7 mmol/l and/or 2-h glucose >= 11·1 mmol/l and/or previously diagnosed with DM; • Hyperglycaemia: FPG > 6·1 mmol/l and/or 2-h glucose > 7·8 mmol/l	• All exposed groups ↑ FPG compared with non-exposed • Late childhood exposed group ↑ hyperglycaemia, and DM *v.* unexposed, similar in severe and less severe famine. In contrast, fetal exposed group affects were limited to severe famine exposure – significant interaction tests, • Prevalence of hyperglycaemia increased if exposed cohort consumed an affluent/western diet/higher BMI at time of glucose assessment	• Large sample size and consideration of effect modification by current diet • Area of current residence used as proxy as area of birth • Likely survivor bias because famine-exposed may have died in childhood
Lu *et al.*, 2020^(80)^	Cohort study. Subset of China Cardiometabolic Disease and Cancer Cohort (4C) study	China medium	• Childhood exposed born January 1949–December 1958 • Fetal and early childhood exposure born January 1959–December 1962 • Unexposed born post famine, 01/1963 − 12/1974	> 40 years at start of follow-up for DM incidence	• 41 148 childhood • 13 195 fetal and early childhood • 23 582 unexposed	• Famine exposure defined by date of birth with respect to famine in 1959–1961 Exclusion: • Missing baseline blood glucose measurements • Diagnosed and screen-detected diabetes at baseline • Missing information on BMI, smoking status, diet habits, physical activity and follow-up glucose measurement • Participants born before 12/1948 excluded.	• FPG • OGTT • DM: FPG > 7 mmol/l and/or 2-h glucose >= 11·1 mmol/l and/or previously diagnosed with DM;	• Childhood exposed group ↑ DM rate *v.* unexposed, similar in fetal exposed group • Age and Sex-adjusted relative risk 1·20 times ↑ in childhood famine exposed group *v.* non-exposed group, but non-significant when further adjusted, and in stratified analyses by non-ideal cardiovascular health metrics, or by sex	• Large sample size • Due to large number of lost to follow-up, poses serious threats to validity • Likely survivor bias because famine-exposed may have died in childhood
Meng *et al.*, 2018^(69)^	Cohort study of diabetes incidence	China High	• Unexposed born October 1962–September 1964 • Fetal exposed born October 1959–September 1962 • Early childhood exposed born October 1956–September 1958	42–48 years at start of follow-up for DM incidence	• 31 363 early childhood • 18 879 fetal • 38 588 unexposed	Inclusion: • Born October 1956–September 1964 (famine was 1959–1961) Exclusion: • Those born transition period October 1958–September 1959 to minimise misclassification • Diagnosed DM since interested in DM incidence • Those with heart disease, stroke or cancer	• Incident DM on clinical register over median of 7·3 years	• 1372 cases of incident DM • After adjustment for age and other confounders, ↑ DM incidence in fetal exposed compared with non-exposed and early childhood exposed combined, no differences by sex. • No evidence of effects in childhood exposed group • Adult obesity and abdominal obesity had additive effects with early MN on diabetes incidence, especially in women, significant interaction	• Study of diabetes incidence over approximately 7 years in mid-life in selected participants of China Kadoorie Biobank • Approximately three times as many prevalent DM cases excluded as incident cases found so difficult to determine overall effect of famine exposure
Portrait *et al.*, 2011^(70)^	Cohort study – Analysis of subset for cumulative % of DM cases	Netherlands Medium	• Fetal and infant (0–1 years) • Childhood (1–5 years) • Pre-adolescent (6–10 years) • Adolescent (11–14 years)	60–76 years	• 278 famine-exposed comprising: 31 aged 0–1 years 102 aged 1–5 years 83 aged 6–10 years 62 aged 11–15 years • 521 unexposed	• Famine exposure: resident in Western Netherlands November 1944–May 1945 • Unexposed: resident in North or East Netherlands same period • Excluded if resident Southern Netherlands due to unclear famine exposure in region	DM as reported in the database	• ↑ Odds of DM in adolescent-exposed females but not males, results adjusted for age current waist circumference (and other factors)	• Exposure and outcome Data derived from Longitudinal Aging Study Amsterdam, nationally representative cohort, but final sample included in this study is not • Note possible error in Table 5 adj OR and crude OR same for females
Stanner *et al.*, 1997^(71)^	Cross-sectional survey	Russia Medium	• Fetal exposed born November 1941–June 1942 • Infant exposed born January 1941–June 1941 • Unexposed group born January 1941–June 1942	52–53 years	• 169 fetal exposed • 192 infant exposed • 188 unexposed	• Famine exposure defined by date of birth with respect to famine in September 1941–January 44 • Unexposed group born during same period but not under siege Exclusion • Died or migrated • DM	• OGTT: Glucose and insulin @0 and 120 min • Fasting proinsulin, C-peptide	• No reported differences between FBG, glucose tolerance, insulin, proinsulin and C-peptide in all groups	• Exposed participants selected from Register of Society of children of the Siege, but final exposed group = 361 out of 1229 eligible from register. • Approximately 68 % remained in Leningrad during approximately 2 years Siege period. • Unclear analysis and comparisons mostly appear to be between fetal and infant exposed rather than non-exposed
Sun *et al.*, 2018^(72)^	Cross-sectional study : subset of China National Health & Retirement Longitudinal Study	China High	• Late childhood exposed born 1949–1952 • Mid-childhood exposed born 1953–1955 • Early childhood exposed born 1956–8 • Fetal and infant exposed born 1959–1962 • Unexposed born 1963–1966	45–62 years	• 7262 comprising: • 1499 late childhood • 1476 mid childhood • 1297 early childhood • 1389 fetal and infant • 1601 unexposed	• Famine exposure defined by date of birth with respect to famine in 1959–1962 • Inclusion = Available FPG and never migrated from province where born. • Participants excluded if lacking key variables or were outliers.	• FPG • HbA1c • Self-report of having been diagnosed with DM • DM: FPG >= 7 mmol/l and/or HbA1c ≥ 6·5 %; • IFG: FPG 5·6–6·9 mmol/l and/or HbA1c 5·7 to 6·4 %; • Hyperglycaemia: if DM or IFG	• After adjustment for sex and famine severity, hyperglycaemia (IFG or DM) ↑ for all ages of famine exposure in females but not males compared with not exposed. Further adjustments do not change this pattern • After adjustment, lower risk of DM (but not hyperglycaemia) in males with early and late childhood exposure compared with unexposed. • Effect in women mostly due to IFG; effect in men due to DM	• Probable overlap of study population with ^(77)^ • Participants and data from baseline data of Nationally representative China Health & Retirement Longitudinal Study • Famine severity calculated by excess death rate for each province • Authors suggest sex difference may be due to different survival of famine between boys and girls
Thurner *et al.*, 2013^(73)^	Nationwide excess risk DM by year of birth	Austria High	All ages: people born between 1917 and 2007; famine exposures were 1918–1919, 1938, 1946–1947	Various ages	325 998 cases of DM (T2DM and T1DM) born 1917–2007 8·3 million Austrian population of same age range from National census	• National database of all pharmacologically treated DM, mostly type 2 • No exclusions because national database	• DM determined clinically and managed with drugs • Reported as % of total population being treated for DM using national census data as denominator	• ↑ Risk of DM for both sexes born during or right after all three famines, indicating risk of fetal exposure. Largest effect in provinces most affected by famine • No excess risk if born just before famines which would have been childhood exposure	• Large database • Analysis adjusted for internal migration flows over time
Van-Abeelen *et al.*, 2012^(74)^	Cohort study: subset of Prospect-EPIC cohort	Netherlands High	0–21 years • Age at Dutch famine exposure grouped into 0–9 years; 10–17 years; 18–21 years • median ages of non-exposed, moderately and severely exposed to famine were 8·3 years, 9·5 years and 10·1 years	49 − 70 years	By self-report/recall: • 3572 unexposed to famine • 2975 moderately exposed • 1290 severely exposed	Inclusion: • Women born before or during Dutch famine 1944–1945 who were part of the EPIC cohort study which collected outcome data Exclusion: • Born after the famine or outside blockaded area 1944–1945 • Missing exposure data • If did not permit access of data from national medical/statistics databases • Already diagnosed DM at enrolment	• DM by self-report or urinary glucose strip during FU and/or diabetes from national hospital register, verified by data from GP/pharmacist	• End of FU 1 Jan 2006 407 incident DM • Adjusted and only age-adjusted ↑ DM risk in moderate and severely exposed *v.* unexposed with significant trend with increased severity of famine • Increased risk and severity trend most prominent in those aged 0–9 years during famine; but effect modification by age group was NS (low sub-group size to detect this)	• Only women studied
Wang *et al.*, 2015^(75)^	Cross-sectional survey - Survey of Prevalence in East China on Metabolic Disease & Risk factors SPECT-China 2014	China High	• Unexposed born after 1963 • Fetal exposed born 1959 − 1962 • Childhood-exposed born 1949–1958 • Adolescent exposed born 1921–1948	Mean ages: • Unexposed < 51 years • Fetal exposed 52–55 years, • Childhood exposed 56–65 years • Adolescent exposed 66–93 years.	• 6897 comprising: • 3053 unexposed • 745 fetal exposed • 1911 childhood exposed • 1188 adolescent exposed	Inclusion: • 18 years old, resident, Chinese citizens, resident in current area > 6 months Exclusion: • Missing laboratory or questionnaire data • < 18 years • Born in 1959 or 1962 (reported in discussion)	• FPG and fasting Insulin • HbA1c • DM defined as FPG ≥ 7·0 mmol/l or HbA1c ≥ 6·5 % and/or previous diagnosis by health professional • Calculations of: • HOMA-IR • HOMA-*β*% • Insulin disposition index (IDI)	• Adjusted DM risk ↑in childhood exposed compared with non-exposed. Effect appears to be due to effect in women with ↑risk in both childhood and adolescent exposed groups and no effect in men. • Exposed child/adolescent women, ↑HOMA-IR, less effect HOMA-*β* % • Exposed child/adolescent males ↓ HOMA- *β* %, no effect HOMA-IR	• Sample representative of E China, stratified for urban/rural and high/low econ status • Indicators of insulin production and glucose metabolism show sex difference
Wang *et al.*, 2016^(78)^	Cross-sectional and incident DM/hyperglycaemia in a subset	China High	• Late childhood born October 1952–September 1954 • Mid childhood born October 1954–September 1956 • Early childhood born October 1956–September 1958 • Fetal and infant born October 1959–September 1961 • unexposed born after the famine 10/1962–09/1964	56·4 (sd 3·3) year	• 7801 in cross-sectional • 1953 late childhood • 1712 mid childhood • 1932 early childhood • 1266 fetal or infant • 938 unexposed • 3100 in cohort analysis 5 years follow-up	Included • Participants in ongoing dynamic cohort exposed to China Famine 1959–1961 Excluded: • Missing FBG or birth date • Participants born during transition periods, October 1958–September 1959 and October 1961–September 1962 excluded	• FPG • HbA1c • DM: FPG >= 7 mmol/l or physician diagnosis • Hyperglycaemia: FPG 6·1–6·9 mmol/l;	• ↑ FPG, HbA1c and hyperglycaemia after childhood famine exposure • ↑risk of DM in late and mid childhood famine-exposed groups; association significant only in women • More severe famine exposure increased risk • No difference in results when stratified by current BMI	• Participants from a cohort of retirees • Severity of famine exposure assessed by region of birth and famine excess mortality rates by regions • Authors suggest lack of increased DM in men may be due to survivor bias since men more likely to die in famine
Wang *et al.*, 2017^(76)^	Cross-sectional subset of Survey of Prevalence in East China on Metabolic Disease & Risk factors SPECT-China 2014–15	China High	• Unexposed born 1963–1974 • Fetal exposed born 1959 − 1962 • Childhood exposed born 1949–1958 • Adolescent and adult exposed born 1926–1948	41–72 years	• 1632 unexposed • 489 fetal-infant exposed • 1140 childhood • 706 adolescent and adult exposed	Inclusion: • Participants from eastern China cohort, representative sampling • Time of exposure to famine 1959–sixty-two defined by date and place of birth Exclusion: • Missing FPG or HbA1c	• FPG • HbA1c • DM: FBG >= 7 mmol/l or HbA1c >= 6·5 %	• ↑DM risk in child or adolescent-adult exposed *v.* unexposed • HbA1c more affected by famine exposure than FPG • Risk increased in areas most severely affected by famine	• Not clear if overlap with those in [70] • Outcome data appears to be same round as [70] • Good control of confounders • Main comparisons between severe and moderate famine exposure but also some comparisons with unexposed born post-famine
Wang *et al.*, 2018^(77)^	Cross-sectional analysis of baseline data of subset of China National Health & Retirement Longitudinal Study	China Medium	• Unexposed born October 1962–September 1964 • Fetal exposed born October 1959–September 1961 • Infant exposed born January 1958–December 1958 • Preschool exposed born January 1956–December 1957	≥ 45 years	• 1536 unexposed • 832 fetal exposed • 519 infant exposed • 1251 preschool exposed	Inclusion: • Participants from a corporate database of retirees • Time of exposure to famine 1959–1961 defined by date and place of birth Exclusion: Missing FPG or HbA1c	• FPG • HbA1c • DM: FBG >= 7 mmol/l or HbA1c >= 6·5 %	• Comparable mean FPG and HbA1c among groups• ↑DM risk in fetal and infant exposed *v.* unexposed• ↑DM risk in overweight/obese fetal exposed *v.* unexposed; not significant in normal weight people	• Note probable overlap with study population in [67] • Large representative cohort and good control of confounders • Analysis focused on fetal exposed group so used postnatal exposed as a control for some analyses, thus masking postnatal exposure effects *v.* unexposed
Woo *et al.*, 2010^(81)^	Cross-sectional study, recruiting 2001–2004)	Hong Kong (Chinese Population) Low	• Childhood not well defined. Mean age at self-reported exposure was 12 (sd = 6) y	≥ 65 years	• 1510 unexposed • 2222 childhood exposed	Inclusion: Participants who were ambulant and living home. Had complete data. Exclusion: Terminal illness and dependent on oxygen	• Self-reported DM, being treated by a doctor	• No DM risk difference between famine exposed *v.* unexposed group	• Famine exposure, but self-reported insufficient food for at one year during childhood. Likely to reporting bias; • DM not well defined • DM not main focus of the study
Zhang *et al.*, 2018^(79)^	Cross-sectional analysis of subset of 2012 Chronic Disease Survey 2012 in Jilin Province	China Medium	• Early childhood exposed born 1956–1958 • Fetal and infant exposed born 1959–1961 • Transitional period born 1962 • Unexposed born 1963–1965	47–55 years	• 1582 early childhood • 1442 fetal and infant • 680 transitional period • 1986 unexposed	Inclusion: • Representative sample from large northeast Chinese database • Individuals born in 1956–1965 with timing of exposure to famine defined by date of birth • Exclusion criteria not mentioned.	• FBG • Blood glucose 2 h after breakfast • DM: FBG >= 7 mmol/l or 2 h glucose >= 11·1 mmol/l or previous diabetes diagnosis • Hyperglycaemia: FBG >= 6·1–6·9 mmol/l or 2 h glucose 7·8 − 11·0 mmol/l	• ↑risk of hyperglycaemia in early childhood exposed women only • ↑ risk of DM in crude analysis in women only exposed during fetal life or childhood; still significant after adjusting for current BMI but only for fetal exposure after adjusting for other confounders	• Large representative sample and control for several confounders • Risk of glucose intolerance after famine exposure significant in women only
Zheng *et al.*, 2012^(82)^	Cross-sectional	China Medium	• Fetally exposed born 1963–1964 • Postnatal exposed born 1957–1958 • Control born 1963–1964	44–51 years	• 1022 fetally exposed • 1344 postnatal • 2674 control	Inclusion • Born 1963–1964 used data from subjects for annual physical examinations from January to December 2008 in Public Health Center of the First Affiliated Hospital of Chongqing Medical University in Chongqing, China; Exclusion • Subjects born in 1959 and 1962	• Dysglycaemia: fasting plasma glucoseX6·1 mmol/l or drug treatment for diabetes	• ↑ Dysglycaemia prevalence in fetally and postnatally exposed groups than in control group	• ↑Metabolic syndrome in both famine groups
Zhou et al,. 2017^(83)^	Cross-sectional	China (Hefei city; E. China) Medium	• Unexposed born October 1962–September 1964 • Fetal exposed born October 1959–September 1961 • Early childhood exposed born September 1956 and October 1958 • Mid childhood exposed born September 1954–October 1956 • Late childhood exposed born September 1952–October 1954	45–60 years	• 381 unexposed • 84 fetal exposed • 160 early childhood exposed • 173 mid childhood exposed • 141 late childhood exposed	Inclusion: • Individuals born in 1956–1965 with timing of exposure to famine defined by date of birth Exclusion criteria: • Participants born during transition periods, October 1958–September 1959 and October 1961–September 1962 excluded.	• FBG • Blood glucose 2 h after breakfast • DM: FBG >= 7 mmol/l or previous self-reported diabetes diagnosed and under anti-diabetic medication	• ↑risk of DM in crude and adjusted analysis in early and mid childhood exposed groups *v.* unexposed group; • ↑ risk of DM in early and mid childhood and degree of high-fat and high-salt dietary pattern in adulthood	• Missing data resulted in 837 being excluded compared with 939 included; • Analyses also conducted by current dietary pattern; • Significant additive interaction for all exposed groups *v.* unexposed with dietary pattern of traditional. Healthy *v.* ↑fat and ↑salt diets

OGTT; oral glucose tolerance test; DM, diabetes mellitus; FBG, fasting blood glucose; FPG, fasting plasma glucose; RPG, random plasma glucose; HbA1c, glycosylated Hb; HOMA, homeostatic model assessments; HOMA-*β*, HOMA *β* cell function; HOMA-IR, HOMA insulin resistance.

### Study design and participants characteristics

There were thirty-two cross-sectional studies,^(18,22,24,27,29,32,34–36,39,42,43,48,51,53,54,57,58,60,66–68,71,72,75–79,81–83)^, twenty-seven cohort studies,^(17,19–21,28,30,31,33,37,41,46,47,49,50,52,55,56,59,61,63–65,69,70,73,74,80)^, of which nineteen were clinical cohorts, mostly comprising short-term follow-up of inpatient malnourished children or adults; three case–control studies^(44,45,62)^, and six intervention trials^(16,23,25,26,38,40)^, not all randomised. Of the total 592 530 participants, most were from the famine studies including one nationwide study with 325 998 participants, mostly non-famine-exposed controls^(73)^. Most other studies had fewer than 100 participants and addressed malnutrition in young children.

### Study quality assessment

Individual studies’ quality scores are shown in Supplementary Data 3. Twelve studies had an overall quality rating considered to be high^(16,59,67–69,72–76,78)^, fifteen studies were rated as medium^(39,44,45,55,58,62,65,66,70,71,77,79,80,82,83)^, seventeen studies were rated as low^(17,18,23–25,27,29,30,35,40,43,46,52,53,57,60,81)^ and the remaining twenty-four as very low^(19–22,26,28,31–34,36–38,41,42,47–51,54,56,61,63,64)^. The main problems resulting in a poor score were not clearly stated study design, undefined sampling strategy, unclear study inclusion and exclusion criteria, low sample size, unclear or absent statistical methods or investigation of confounders or lack of appropriate control/healthy comparison groups. These problems were mainly found in older studies done at a time of different expectations for study design and presentation but many of these studies appear otherwise carefully conducted and contain valuable information for this review. It should be noted that similar data found in some of these studies is unlikely to be collected in future since the studies used invasive techniques in children which would not be approved by most modern ethics committees.

### Definition of the malnutrition exposure

The definition of childhood severe malnutrition has varied over the publication dates of included studies from clinical definitions of kwashiorkor and marasmus, comparison with various different child growth standards, and more recently, the current WHO definition of severe acute malnutrition based on the 2005 growth standards or oedema^(84)^. While there may be minor differences between definitions, we believe these likely reflect broadly the same clinical conditions across time and have therefore considered the definitions of childhood clinical malnutrition together. The more important difference for effects on the pancreas appears to be whether or not the severe acute malnutrition involved oedema, that is, kwashiorkor. Adult malnutrition in the included papers has generally been caused by low BMI and/or a clinical diagnosis of AN. For the studies of long-term consequences of childhood famine exposure, there were no assessments of malnutrition at the time so exposure was defined by date and place of residence at the time.

### Definitions of exocrine and endocrine pancreas outcomes

Tests used in the included studies of exocrine pancreas function fit into two main groups. Some earlier work measured enzymes in duodenal juice collected with a catheter, both basally and after stimulation with secretin or cholecystokinin, whereas recent studies were less invasive and collected only faeces or serum; this makes it hard to compare results. Studies using catheters and collecting duodenal fluid before and after simulation measured various enzymes, including amylase, lipase, phospholipase, trypsin and chymotrypsin, as well as bicarbonate and electrolytes^(19,22–26,28,48,49)^. Recent studies assessed faecal elastase, which is low in pancreatic insufficiency^(16,17,27,50)^, or blood levels of enzymes such as trypsinogen, amylase or lipase which may be either high or low in pancreatic disorders^(18,20,21)^. One study measured pancreas head size in children using ultrasound^(21)^ and another measured d-xylose and triglyceride absorption^(50)^.

As for exocrine pancreas tests, the older and more recent literature generally use different tests for endocrine function, although this is partly driven by the fact that much of the more recent literature is from post-famine studies which, with their very large sample sizes, use mostly simple tests or diagnoses from clinical databases. In some studies, the main outcome was fasting blood or plasma glucose^(29,38,44–46,72,76,77,79,80,82,83)^. One study had only random plasma glucose^(67)^. HbA1c was measured mainly but not exclusively in the large famine studies^(29,65,72,75–78)^. Some of these large studies used previously clinically diagnosed diabetes from national or other large databases^(59,69,70,73,74,80–83)^. Oral glucose tolerance tests (OGTT)^(30,31,33,35,39,40,43–45,58,64–66,68,71,80,83)^ were frequently conducted as were intravenous glucose tolerance tests (IVGTT)^(30–34,36,37,41,42,47,51,60,63)^. These generally followed similar standard protocols. Some researchers measured insulin as well as glucose in these tests. Researchers rarely used these tolerance tests to diagnose diabetes but were interested in various glucose metabolism indicators calculated from the glucose and insulin levels during the tests, including AUC, insulin : glucose ratios and glucose disappearance rate. Homeostatic model assessment indices were rarely calculated^(46,75)^. Several studies investigated glucose metabolism after injection or perfusion with glucagon^(43,47,56)^, insulin^(46,55)^ or arginine^(51,54,55)^. There were single studies using each of the following methods: a standard meal as the glucose challenge^(57)^, a euglycaemic hyperinsulinaemic clamp method^(61)^ and only 24-h C-peptide excretion^(53)^.

### Exocrine pancreas function during and after malnutrition in children and adults

Most papers addressing exocrine pancreas function were from hospital-based studies of acute malnutrition in young children, at admission, during treatment or at hospital discharge; two papers^(24,28)^ also included some children longer after discharge (Table 1). Among the seven studies which measured duodenal enzymes, most both before and after pancreas stimulation^(19,22–26,28)^, all but one^(22)^ found decreases in several enzymes (amylase, lipase, trypsin, and chymotrypsin). Short-term nutritional therapy in hospital usually increased enzyme levels but not always to levels of well-nourished controls. Discrepancies among studies may be due to small sample sizes, differences in patient populations and the duration and type of nutritional or other therapy.

Three studies^(16,17,27)^, two from the same research group, used faecal elastase as the exocrine pancreas marker. The study in Indonesia^(27)^, which was concerned mainly with diarrhoea and did not clearly define malnutrition, found no differences in elastase between malnourished children at hospital admission and well-nourished children. The studies in Malawi^(16,17)^ included no well-nourished children but investigated changes over time during therapy among malnourished children; children with oedematous malnutrition had lower faecal elastase than those with non-oedematous and generally did not recover within a month of nutritional therapy. In all these studies, there is a concern that diarrhoea, which is common among children hospitalised with malnutrition, could interfere with the use of faecal elastase as a marker of pancreas function.

Four studies, all with fairly large sample sizes, measured pancreatic enzymes in blood or serum.^(17,18,20,21)^. Circulating trypsinogen was higher in children with oedematous compared with non-oedematous malnutrition^(17)^, and in malnourished children than in healthy controls^(20)^. In one study, serum trypsinogen was not associated with malnutrition but was increased in children with gastroenteritis^(18)^. Serum amylase and lipase, as well as pancreas head size, were low in malnourished children at hospital admission compared with healthy controls and increased during treatment^(21)^.

There is limited information about exocrine pancreas function during or after malnutrition occurring in adulthood (Table 2). There was no difference in faecal elastase among ten AN patients before and after nutritional recovery; this study also found normal d-xylose and triglyceride absorption^(50)^. Two papers from the same group in India studied possibly overlapping small numbers of malnourished adults and controls^(48,49)^. Before nutritional therapy, malnourished adults had steatorrhoea and low duodenal juice contents of trypsin and lipase basally and post-stimulation with cholecystokinin (pancreozymin) and secretin; amylase content was low only post-stimulation. After nutritional therapy, lipase and amylase differed little from values in healthy controls both before and after stimulation while trypsin remained low.

### Endocrine pancreas function

#### Studies in young children

This group comprises the bulk of the included papers but only one was scored as medium quality^(39)^ and the rest were rated as low or very low quality. Fasting blood or plasma glucose (not always distinguished in the papers and referred to here as FBG), was generally unaffected by acute malnutrition^(33–35,37,38,47)^ but decreased FBG was also reported^(29,36,46)^. HbA1c was higher in children with kwashiorkor or marasmus compared with controls and FBG and HbA1c were inversely associated^(29)^. Altered fluid balance, especially among children with oedematous malnutrition, and the more acute time frame represented by FBG than by HbA1c may have contributed to an apparently anomalous negative correlation between FBG and HbA1c.

Slow glucose disappearance rates during OGTT or IVGTT were commonly seen at hospital admission in malnourished children, especially those with kwashiorkor^(32–34,37,41)^ but not always in those with marasmus^(33)^. One study^(32)^ suggested that lack of insulin was not the reason for slow glucose disappearance since insulin infusion in four children with kwashiorkor did not increase the glucose disappearance rate. Another study^(33)^ found that glucose disappearance rate in kwashiorkor patients was negatively correlated with blood fatty acids which suggest a role for insulin resistance. However, a small study which used stable isotopes to investigate glucose metabolism found no evidence for hepatic or peripheral insulin resistance but did note that glucose clearance was positively correlated with plasma albumin^(39)^. Nutritional therapy generally normalised glucose disappearance rate^(33,37,41)^ although one study found this was still slow in children 6–12 years post-hospitalisation for kwashiorkor^(42)^.

Fasting plasma insulin in different studies of kwashiorkor or marasmus was variable, likely reflecting variable populations and small sample sizes^(33,35,38)^. Insulin release during an OGTT or IVGTT, measured either as plasma levels or in relation to glucose, was generally low for children admitted to hospital mainly with kwashiorkor but sometimes also with non-oedematous malnutrition^(30,31,34,39–41)^, although one study found high peak insulin during an OGTT in malnourished children^(35)^. Insulin levels increased in the short term with nutritional therapy but were not always restored to normal even months after admission^(30,33,34,37,38)^, although the overall patterns of response normalised^(31)^. Interestingly, in a series of studies by the same research group with overlapping patient populations^(30,31,40)^, insulin/glucose AUC was more reduced and patterns of response more abnormal compared with controls during an OGTT than in an IVGTT, and a pattern of delayed insulin secretion in malnutrition was common; both these could indicate that part of the impaired insulin response was due to factors in the intestine. One study measured plasma glucagon and found low fasting levels in children admitted to hospital for malnutrition^(38)^.

Five studies investigated endocrine pancreas function using OGTT or IVGTT in participants several years after they were hospitalised for malnutrition in early childhood^(30,42–45)^. Ten years post-kwashiorkor, there was no difference compared with sibling controls in peak insulin or insulin/glucose AUC in an IVGTT^(30)^. There were two small studies from the same malnutrition unit in Uganda of children about 10 years after they recovered from kwashiorkor. One found a slower glucose disposal rate during an IVGTT in recovered malnourished children compared with controls^(42)^. The other study found lower fasting insulin in recovered kwashiorkor patients but normal insulin response in an OGTT and with a glucagon stimulus to elicit maximal insulin release^(43)^. More recent studies with larger sample sizes found larger differences in glucose metabolism of malnutrition survivors compared with controls. Jamaican adults who had experienced marasmus in early childhood had lower insulin secretion and poorer glucose tolerance in an OGTT compared with kwashiorkor survivors or not previously malnourished controls^(44)^. Among young adult Mexican male survivors of childhood malnutrition, plasma glucose concentration and AUC in an OGTT were higher than in not previously malnourished controls only after controlling for BMI, age and birth weight, whereas plasma insulin was higher both with and without controlling for these variables and the difference between cases and controls was greater in those with higher BMI^(45)^. A recent study in 1080 Chilean adults found higher glycaemia at age 22–28 years in those who were wasted or at risk of wasting at 12 months (WLZ score < –2 or < –1), including after adjustment for confounders including birth weight and gestational age. Those who were underweight (WAZ-score < –2) at 12 months had evidence of increased glycaemia in unadjusted but not adjusted analysis but increased insulin sensitivity when assessed using single point insulin sensitivity estimate but not homeostatic model assessment-insulin resistance^(46)^.

#### Endocrine pancreas function after malnutrition in later life

Of fifteen papers on endocrine pancreas function after malnutrition experienced in later childhood or adulthood, twelve are about AN patients, only one of which included males^(59)^, two are about Indian adults and one is about African adults (Table 2). The quality scores for the majority in this group were classified as low or very low, with the exception of a study in Sweden^(59)^ which utilised a national register with long-term follow-up and was rated as high quality, plus four studies rated as medium quality: one from the USA^(58)^, a case–control study in malnourished Indian adults^(62)^, a cohort study in African malnourished adults^(65)^ and a study in Japanese women with AN before and 5 months after treatment^(55)^. When patients were admitted to hospital with AN, there was a fairly consistent finding of abnormal, that is, low or delayed, insulin production during OGTT, IVGTT, arginine or glucagon infusion or after a meal^(51,52,54–56,61,64)^. Glucose metabolism during these tests was more variable: there was often poor glucose tolerance, as might be expected from the low insulin production^(51,52,64)^, but others found normal responses^(55)^. Plasma glucagon was generally not different between AN patients and controls^(51,54)^ although one study found abnormal patterns of glucagon changes during an OGTT^(52)^, and another found low glucagon after insulin-induced hypoglycaemia but not after arginine infusion^(55)^. One study found that 24-h urinary excretion of C-peptide did not differ between AN patients and controls^(53)^.

Several studies examined AN patients after weight recovery either just before discharge from care or several years later. Not all studies included controls so it is difficult to determine whether normal pancreas endocrine function was achieved following weight regain. Insulin production and glucose tolerance often improved compared with admission results by the time, usually after several months, AN patients had gained sufficient weight to be discharged^(61,64)^, but did not always reach normal levels^(52,56)^. A study of AN patients who had recovered weight, but which provided no information on time since diagnosis, found continued impairments in insulin production but heightened insulin sensitivity resulting in similar glucose responses following a test meal^(57)^. Insulin production remained low and glucose tolerance impaired 8–10 years after AN diagnosis in those who remained low weight but not in those who had achieved normal weight^(58)^. The incidence of diabetes diagnoses on a Swedish national register was lower among former AN patients than among the general population but not different from sibling controls; the low incidence among those with prior AN may have been related to their very low incidence of overweight as adults but the study was not designed to control for this^(59)^.

In a study of malnourished Indian adults, in which malnutrition duration and severity were not well defined and there were no well-nourished controls, insulin increase was slow but prolonged in an IVGTT, glucose disposal rate was low and both glucose and insulin responses to arginine infusion were blunted; all responses improved after 2–4 months of nutritional therapy^(63)^. A case–control study of tropical pancreatitis in Indian adults found that 13·5 % of the patients had diabetes, based on fasting and postprandial blood glucose levels, and that weight loss appeared a consequence, not a cause, of the impaired pancreas function^(62)^. A cohort study in African adults reported that malnutrition associated mainly with HIV or tuberculosis infection 7–12 years previously was later associated with lower insulin levels in an OGTT in men but not in women^(65)^.

#### Studies in adults exposed to famine in childhood

The famine follow-up studies (Table 3) represent the largest amount of information on long-term outcomes of childhood malnutrition, with by far the largest sample sizes, with generally robust statistics, and when participants were in middle age when diabetes is more common than at younger ages. The famine studies were the majority of studies rated good or medium in the quality assessments. The drawback of the famine studies is that the diagnosis of prior malnutrition is based on date and place of birth so it cannot generally account for local differences in famine exposure or individual or family response to famine, although one study asked participants what they recalled of their famine experience^(74,81)^. Another concern is that, particularly in the Chinese famine of 1959–1961 which was prolonged and had high mortality, there is likely to be a survivor bias, and this may have had a sex difference, that is, boys may have had higher mortality than girls^(72,78,80–83)^.

Eleven of the eighteen included studies were from China, were done by different research teams and together included data from six representative cohorts (one with two publications from the same group with different purposes^(75,76)^ and another one with two publications by different research groups^(72,77)^). Methods were similar in that famine exposure was determined by birth location and date with respect to the 1959–1961 famine. Most studies also examined fetal famine exposure which is not the concern here. There were differences among studies regarding the sex and postnatal age for which famine exposure carried the greatest risk of hyperglycaemia (assessed by fasting blood glucose and/or HbA1c) or diabetes: all ages from early to late childhood^(75,76,80,82,83)^, all ages but only in women^(72,78)^, early childhood in women only^(79)^, infancy only^(77)^ or late childhood only^(68,81)^. Although both men and women were at increased risk of high fasting plasma glucose or HbA1c, if exposed prenatally, *β*-cell function, indicated by homeostatic model assessment-β, seemed to be the major problem in men, whereas it was insulin resistance, indicated by homeostatic model assessment-insulin resistance, in women^(75)^. Women exposed to famine at any stage of childhood had an increased prevalence of hyperglycaemia but not of diabetes, whereas men in the study had no difference in hyperglycaemia but lower prevalence of diabetes^(72)^. In another study that examined sex differences, this was only observed for risk of composite metabolic syndrome, with similar risks among men and women for hyperglycaemia^(82)^. There appears to be an interaction between famine exposure and diet or BMI at the time of glucose assessment: being overweight or currently eating a Western style rather than traditional Chinese diet increased the risk of hyperglycaemia or diabetes after childhood famine exposure^(68,77,83)^. One cohort study investigated the incidence of clinically diagnosed diabetes over about 7 years in middle age^(69)^. The incidence was increased among those exposed to famine in utero but not those exposed in childhood and was aggravated by adult abdominal obesity; however, about three times as many cases of prevalent diabetes were excluded from the study as were found to have incident diabetes so it is difficult to determine the overall effect of famine exposure.

The Dutch famine of 1944–1945 has been greatly studied for long-term effects of in utero exposure but less so for postnatal exposure. Famine exposure during adolescence, and to a borderline extent earlier in childhood, increased risk of a later diabetes diagnosis in women but not in men^(70)^. A study which included only women^(74)^ found an increased risk of later diabetes by self-report or clinical diagnosis if exposed to famine at any time during childhood and the risk was increased if women reported their famine exposure as severe *v.* moderate.

Famines in Bangladesh, Hong Kong (on Chinese population), Nigeria, Russia and Austria are represented by one paper each. Early childhood famine exposure in Bangladesh did not affect fasting glucose or glucose response in an OGTT^(66)^. In Nigeria, childhood famine exposure had no effect on random blood glucose; unfortunately, the study’s recruitment method precluded use of other tests of glucose metabolism^(67)^. In Hong Kong, self-reported childhood famine exposure was not associated with DM risk^(81)^. Childhood famine in Russia was not associated with glucose, insulin, proinsulin or C-peptide in adulthood^(71)^. Analysis of a large Austrian national database which included people of a wide age range covering fetal or childhood exposure to several 20^th^ century famines found clear evidence of increased risk of fetal famine exposure but not of childhood exposure^(73)^. It is unclear why these studies from other countries differ from the general findings of ongoing impaired endocrine pancreas function seen in the Chinese and Dutch famine studies, but differences in famine experience and mortality and in later environment and diet could be important.

## Discussion

There is considerably more research on how postnatal malnutrition affects endocrine than exocrine pancreas function. This likely reflects the high prevalence of diabetes globally and its serious health consequences but is in part because there are fewer non-invasive tests of exocrine than endocrine pancreas function available. Some earlier work on exocrine pancreas function used catheters to collect duodenal juice, but such tests are unlikely now to be considered ethically justified for research and the earlier work was of generally poor quality due to low participant numbers, inadequate statistics and consideration of confounding and high risk of selection bias.

Overall, while there are differences among studies of exocrine pancreas function, it seems that secretion of many pancreatic enzymes is reduced in acute childhood malnutrition. The several papers reporting steatorrhoea suggest that this reduced enzyme secretion may have important functional consequences which, through impaired nutrient absorption, could have contributed to the malnutrition in the first place and would very likely exacerbate it. In most cases, nutritional therapy improved enzyme secretion although not always to control levels, possibly because of varying durations and quality of the therapy. One trial which investigated adding enzyme therapy to nutrition^(16)^ found no additional benefits; this was a recent study using current WHO nutritional therapy guidelines which likely provide better nutritional support than was available in earlier studies. Our results are consistent with a previous review^(6)^ which found an association between malnutrition and decreased exocrine pancreas function but could not determine causality.

Regarding endocrine pancreas function, there seem to be prolonged impairments in insulin production among people severely malnourished in childhood or adulthood but these are most profound in people who remain malnourished^(46,47)^. Adults in LMIC recruited when malnourished may have been so through much of their lives^(48,49)^ and long-term impairments in AN patients are greatest in those who remain malnourished^(55,57)^. It would be interesting to investigate insulin in people in LMIC of previously good adult nutritional status who first became malnourished in adulthood. However, adult-onset malnutrition often follows serious infections, for example, with HIV or tuberculosis, or cancers so these factors confound the situation. AN remains the most common cause of severe malnutrition resulting mainly from low dietary intake. Since there appear similarities between observations in AN patients, people exposed to famine in childhood^(74,78)^ and adults in LMIC, this suggests that it is malnutrition itself, rather than only the accompanying infections, environmental enteropathy and other aspects of living in poverty, that influence pancreatic insulin production^(55,64,65,70,74)^. Furthermore, since AN normally occurs in people who were previously adequately nourished in high-income countries, the results from AN patients suggest that the direction of causality is from malnutrition to impaired pancreas function, although the opposite direction of causality, with pancreas disease causing malnutrition, may also contribute^(62)^.

The mechanisms whereby malnutrition may result in long-term effects on the pancreas are unclear and the studies included in this review provide little information, in part because the more recent and high-quality studies have been mainly large ones investigating the epidemiology of glucose metabolism in famine survivors. Some studies in India have investigated pancreas calcification as the mechanism of the impaired function; such reports contributed to the earlier WHO classification of fibrocalculus pancreatic diabetes^(11)^ but most such studies in the present review were excluded because the main exposure was not prior nutritional status. Environmental enteropathy, which is common in low-income countries and often associated with malnutrition^(85)^, may have contributed to impaired glucose tolerance in studies of acute malnutrition. Evidence for this comes from studies showing delayed insulin responses or larger effects on the insulin response to OGTT than to IVGTT which bypasses the gut. Possible mechanisms of intestinal epithelial involvement include delayed or reduced glucose absorption^(86)^ during an OGTT and reduced insulin production because of low incretin production by enterocytes^(87)^.

Most abnormalities in endocrine pancreas function of severely malnourished people seem to improve in the short term with the treatment of the malnutrition; recovery of exocrine pancreas functions after malnutrition has been less studied. There is limited information about long-term pancreas function outside the famine studies. Several of those studies suggest early insults may interact with later diet and illness^(68,77)^.This is in keeping with the capacity load model of chronic disease^(88)^ in which damage to a physiological capacity, for example, pancreas functions, earlier in life is most likely to result in health problems if in later life there is a greater load on the system, for example, due to overweight or consumption of a diet high in sugar. Differing prevalence of adult obesity in men and women, in addition to potential sex differences in survival from malnutrition, may contribute to the variable sex differences seen in some of the famine follow-up studies.

### Strengths and limitations of the review

A strength of the review is that it included a large number of studies from many countries of varying income levels and with multiple study designs and participant characteristics. The overall similarity in results from very different studies, that is, clinical malnutrition in young children, AN in older children and adults and follow-up of famine studies, lends credence to the findings. At least two of the authors reviewed all the included studies. The review has limitations resulting from the heterogeneity of data from varying methodologies, settings and populations enrolled which also precluded being able to conduct any meaningful meta-analyses. Many of the included studies were of poor quality with small sample sizes, poorly defined populations and unclear statistics. We did not analyse the findings of only good quality studies separately since this would have excluded too many that provided data not available elsewhere. Many of the early studies were conducted to define the aetiology and biology of kwashiorkor *v.* marasmus so we were not addressing our interests specifically. Similarly, much research on AN was not specifically investigating pancreas function. The techniques used in older compared with newer studies differed and were not always validated, so results are hard to compare. Authors of some of the famine studies were interested in prenatal famine exposure, so they included postnatal exposure as a control for that. The absence of an original aim to investigate postnatal malnutrition and pancreas function could have meant that, even if the study contained data relevant to our search, the title and abstract might not have mentioned it so it would have been missed at the first level of the search; this may explain why a large proportion of the included studies were actually located from the reference lists of other articles found. In addition, we did not include studies where the population was selected based on diabetes because this could have resulted in bias in relation to our aims; for example, studies looking at malnutrition as one of many risk factors for diabetes in a population may have included it in the abstract only if the association was statistically significant, for example, Fekadu *et al.* ^(89)^

### Conclusion

Much of the world is currently facing a double burden of under- and over-nutrition in which there is an increasing prevalence of overweight, diabetes and other chronic diseases but an ongoing high prevalence of malnutrition, both in young children and in older individuals with severe infections. There is a need for a better understanding of how these conditions interact in order to improve prevention and treatment of chronic conditions. This review suggests that malnutrition at any postnatal age can have both acute and long-term adverse effects on pancreas function so that diabetes treatments should consider insulin production as well as insulin resistance. Currently, the common first-line pharmacological treatment for diabetes in many settings, including low-income ones where detailed metabolic investigations are often not possible, is metformin which acts primarily on insulin resistance; however, it may not be the best treatment in populations where low insulin production is a major concern^(90)^. The similarity of findings from very different populations, including children living in poor environments, adults with malnutrition secondary to severe infections, AN patients and famine survivors, suggests that it is malnutrition itself which can result in impaired pancreas functions. If infection-mediated malnutrition has life-long impacts on diabetes risk, this provides added impetus to prevent and treat this malnutrition beyond achieving favourable outcomes of the original infection, for example, tuberculosis or HIV. More well-designed research with clearly defined populations, adequate sample sizes, consideration of the sexes separately and using robust current techniques to determine the contribution of low insulin production or increased insulin resistance, is needed in order to understand both the epidemiology and mechanisms of interactions between malnutrition and pancreas functions.

## Supplementary material

For supplementary material accompanying this paper visit https://doi.org/10.1017/S0007114522001404. click here to view supplementary material

## References

[1] Ashworth A , Khanum S , Jackson A , et al. (2003) Guidelines for the Inpatient Treatment of Severely Malnourished Children. Geneva: WHO.

[2] Dalvi PS , Yang S , Swain N , et al. (2018) Long-term metabolic effects of malnutrition: Liver steatosis and insulin resistance following early-life protein restriction. PLOS ONE 13, e0199916. 29965973 10.1371/journal.pone.0199916 PMC6028108

[3] Ibrahim MK , Zambruni M , Melby CL , et al. (2017) Impact of childhood malnutrition on host defense and infection. Clin Microbiol Rev 30, 919–971. 28768707 10.1128/CMR.00119-16 PMC5608884

[4] Calkins K & Devaskar SU (2011) Fetal origins of adult disease. Curr Probl Pediatr Adolesc Health Care 41, 158–176. 21684471 10.1016/j.cppeds.2011.01.001 PMC4608552

[5] Dolenšek J , Pohorec V , Rupnik MS , et al. (2017) Pancreas Physiology. Challenges in Pancreatic Pathology. Rijeka, Croatia: IntechOpen.

[6] Bartels RH , van den Brink DA , Bandsma RH , et al. (2018) The relation between malnutrition and the exocrine pancreas: a systematic review. J Pediatr Gastroenterol Nutr 66, 193–203. 28991838 10.1097/MPG.0000000000001769

[7] World health organization (2020) Diabetes. Geneva: WHO.

[8] Leitner DR , Fruhbeck G , Yumuk V , et al. (2017) Obesity and type 2 diabetes: two diseases with a need for combined treatment strategies – EASO can lead the way. Obes Facts 10, 483–492. 29020674 10.1159/000480525 PMC5741209

[9] World Health Organization (1965) Diabetes Mellitus: Report of a WHO Expert Committee. Geneva: WHO. 4953441

[10] World Health Organization (1980) WHO Expert Committee on Diabetes Mellitus. Geneva: WHO.

[11] World Health Organization Study Group on Diabetes Mellitus (1985) Diabetes Mellitus: Report of a WHO Study Group. Geneva: WHO.

[12] Bavuma C , Sahabandu D , Musafiri S , et al. (2019) Atypical forms of diabetes mellitus in Africans and other non-European ethnic populations in low- and middle-income countries: a systematic literature review. J Glob Health 9, 020401. 31673335 10.7189/jogh.09.020401 PMC6818125

[13] Grey K , Gonzales GB , Abera M , et al. (2021) Severe malnutrition or famine exposure in childhood and cardiometabolic non-communicable disease later in life: a systematic review. BMJ Glob Health 6, e003161. 10.1136/bmjgh-2020-003161 PMC7949429 33692144

[14] von Elm E , Altman DG , Egger M , et al. (2007) The Strengthening the Reporting of Observational Studies in Epidemiology (STROBE) Statement: guidelines for reporting observational studies. Bull World Health Org 85, 867–872. 18038077 10.2471/BLT.07.045120 PMC2636253

[15] Schulz KF , Altman DG , Moher D , et al. (2010) CONSORT 2010 Statement: updated guidelines for reporting parallel group randomised trials. BMJ 340, c332. 20332509 10.1136/bmj.c332 PMC2844940

[16] Bartels RH , Bourdon C , Potani I , et al. (2017) Pancreatic enzyme replacement therapy in children with severe acute malnutrition: a randomized controlled trial. J Pediatr 190, 85–92.e82. 28912050 10.1016/j.jpeds.2017.07.013

[17] Bartels RH , Meyer SL , Stehmann TA , et al. (2016) Both exocrine pancreatic insufficiency and signs of pancreatic inflammation are prevalent in children with complicated severe acute malnutrition: an observational study. J Pediatr 174, 165–170. 27178623 10.1016/j.jpeds.2016.04.013

[18] Briars GL , Thornton SJ , Forrest Y , et al. (1998) Malnutrition, gastroenteritis and trypsinogen concentration in hospitalised Aboriginal children. J Paediatr Child Health 34, 69–73. 9568946 10.1046/j.1440-1754.1998.00157.x

[19] Danus O , Urbina AM , Valenzuela I , et al. (1970) The effect of refeeding on pancreatic exocrine function in marasmic infants. J Pediatr 77, 334–337. 5431213 10.1016/s0022-3476(70)80347-x

[20] Durie PR , Forstner GG , Gaskin KJ , et al. (1985) Elevated serum immunoreactive pancreatic cationic trypsinogen in acute malnutrition: evidence of pancreatic damage. J Pediatr 106, 233–238. 3968610 10.1016/s0022-3476(85)80293-6

[21] El-Hodhod MA , Nassar MF , Hetta OA , et al. (2005) Pancreatic size in protein energy malnutrition: a predictor of nutritional recovery. Eur J Clin Nutr 59, 467–473. 15536474 10.1038/sj.ejcn.1602053

[22] Keni S , Jain MK , Mehra R , et al. (1995) Impaired pancreatic bicarbonate secretion in chronic malnutrition. Indian Pediatr 32, 323–329. 8613287

[23] Mehta HC , Saini AS , Singh H , et al. (1984) Pancreatic functions in marasmic children: effect of dietary therapy. Indian Pediatr 21, 149–153. 6432690

[24] Sauniere JF , Sarles H , Attia Y , et al. (1986) Exocrine pancreatic function of children from the Ivory Coast compared to French children. Effect of kwashiorkor. Dig Dis Sci 31, 481–486. 3009110 10.1007/BF01320311

[25] Sauniere JF & Sarles H (1988) Exocrine pancreatic function and protein-calorie malnutrition in Dakar and Abidjan (West Africa): silent pancreatic insufficiency. Am J Clin Nutr 48, 1233–1238. 3189210 10.1093/ajcn/48.5.1233

[26] Thompson MD & Trowell HC (1952) Pancreatic enzyme activity in duodenal contents of children with a type of kwashiorkor. Lancet 1, 1031–1035. 14928553 10.1016/s0140-6736(52)90692-2

[27] Widodo AD , Timan IS , Bardosono S , et al. (2016) Pancreatic exocrine insufficiency in malnourished children and those with persistent diarrhoeae. Asia Pac J Clin Nutr 25, S57–S61. 28027633 10.6133/apjcn.122016.s3

[28] Barbezat GO (1967) The exocrine pancreas and protein-calorie malnutrition. S Afr Med J 41, 84. 6020162

[29] Adegbenro SA , Dada OA , Olanrewaju DM , et al. (1991) Glycosylated haemoglobin levels in children with protein-energy malnutrition. Ann Trop Paediatr 11, 337–341. 1721790 10.1080/02724936.1991.11747525

[30] Becker DJ , Pimstone BL , Hansen JD , et al. (1971) Insulin secretion in protein-calorie malnutrition. I. Quantitative abnormalities and response to treatment. Diabetes 20, 542–551. 5565001 10.2337/diab.20.8.542

[31] Becker DJ , Pimstone BL , Hansen JD , et al. (1972) Patterns of insulin response to glucose in protein-calorie malnutrition. Am J Clin Nutr 25, 499–505. 4623275 10.1093/ajcn/25.5.499

[32] Bowie MD (1964) Intravenous glucose tolerance in Kwashiorkor and Marasmus. S Afr Med J 38, 328–329. 14145116

[33] Hadden DR (1967) Glucose, free fatty acid, and insulin interrelations in kwashiorkor and marasmus. Lancet 2, 589–592. 4166829 10.1016/s0140-6736(67)90740-4

[34] James WP & Coore HG (1970) Persistent impairment of insulin secretion and glucose tolerance after malnutrition. Am J Clin Nutr 23, 386–389. 5441174 10.1093/ajcn/23.4.386

[35] Garg SK , Marwaha RK , Ganpathy V , et al. (1989) Serum growth hormone, insulin and blood sugar responses to oral glucose in protein energy malnutrition. Trop Geogr Med 41, 9–13. 2503912

[36] Slone D , Taitz LS & Gilchrist GS (1961) Aspects of carbohydrate metabolism in Kwashiorkor. Br Med J 1, 32–34. 20789028 10.1136/bmj.1.5218.32 PMC1952741

[37] Prinsloo JG , De Bruin EJ & Kruger H (1971) Comparison of intravenous glucose tolerance tests and serum insulin levels in kwashiorkor and pellagra. Arch Dis Child 46, 795–800. 4942942 10.1136/adc.46.250.795 PMC1647919

[38] Robinson HM & Seakins A (1982) Fasting pancreatic glucagon in Jamaican children during malnutrition and subsequent recovery. Pediatr Res 16, 1011–1015. 6818514 10.1203/00006450-198212000-00008

[39] Spoelstra MN , Mari A , Mendel M , et al. (2012) Kwashiorkor and Marasmus are both associated with impaired glucose clearance related to pancreatic beta-cell dysfunction. Metabolism 61, 1224–1230. 22386944 10.1016/j.metabol.2012.01.019

[40] Becker DJ , Mann MD , Weinkove E , et al. (1975) Early insulin release and its response to potassium supplementation in protein-calorie malnutrition. Diabetologia 11, 237–239. 807496 10.1007/BF00422328

[41] Becker DJ , Pimstone BL & Hansen JDL (1975) The relation between insulin secretion, glucose tolerance, growth hormone, and serum proteins in proteincalorie malnutrition. Pediatr Res 9, 35–39.

[42] Cook GC (1967) Glucose tolerance after kwashiorkor. Nature 215, 1295–1296. 6052737 10.1038/2151295a0

[43] Kajubi SK (1972) The endocrine pancreas after kwashiorkor. Am J Clin Nutr 25, 1140–1142. 4563843 10.1093/ajcn/25.11.1140

[44] Francis-Emmanuel PM , Thompson DS , Barnett AT , et al. (2014) Glucose metabolism in adult survivors of severe acute malnutrition. J Clin Endocrinol Metab 99, 2233–2240. 24517147 10.1210/jc.2013-3511

[45] Gonzalez-Barranco J , Rios-Torres JM , Castillo-Martinez L , et al. (2003) Effect of malnutrition during the first year of life on adult plasma insulin and glucose tolerance. Metabolism 52, 1005–1011. 12898465 10.1016/s0026-0495(03)00151-3

[46] Pereyra I , Lopez-Arana S & Horta BL (2021) Undernutrition and suboptimal growth during the first year are associated with glycemia but not with insulin resistance in adulthood. Cad Saude Publica 37, e00120320. 34346980 10.1590/0102-311X00120320

[47] Milner RDG (1971) Metabolic and hormonal responses to glucose and glucagon in patients with infantile malnutrition. Pediatric Research 5, 33–39.

[48] Tandon BN , George PK , Sama SK , et al. (1969) Exocrine pancreatic function in protein--calorie malnutrition disease of adults. Am J Clin Nutr 22, 1476–1482. 5350763 10.1093/ajcn/22.11.1476

[49] Tandon BN , Banks PA , George PK , et al. (1970) Recovery of exocrine pancreatic function in adult protein-calorie malnutrition. Gastroenterology 58, 358–362. 5437989

[50] Martinez-Olmos MA , Peino R , Prieto-Tenreiro A , et al. (2013) Intestinal absorption and pancreatic function are preserved in anorexia nervosa patients in both a severely malnourished state and after recovery. Eur Eat Disord Rev 21, 247–251. 23389861 10.1002/erv.2223

[51] Blickle JF , Reville P , Stephan F , et al. (1984) The role of insulin, glucagon and growth hormone in the regulation of plasma glucose and free fatty acid levels in anorexia nervosa. Horm Metab Res 16, 336–340. 6386641 10.1055/s-2007-1014785

[52] Kumai M , Tamai H , Fujii S , et al. (1988) Glucagon secretion in anorexia nervosa. Am J Clin Nutr 47, 239–242. 3277371 10.1093/ajcn/47.2.239

[53] Wallensteen M , Ginsburg BE , Persson B , et al. (1991) Urinary C-peptide excretion in obese and anorectic children. Acta Paediatr Scand 80, 521–526. 1872175 10.1111/j.1651-2227.1991.tb11896.x

[54] Sizonenko PC , Rabinovitch A , Schneider P , et al. (1975) Plasma growth hormone, insulin, and glucagon responses to arginine infusion in children and adolescents with idiopathic short stature, isolated growth hormone deficiency, panhypopituitarism, and anorexia nervosa. Pediatr Res 9, 733–738. 1105371 10.1203/00006450-197509000-00010

[55] Fujii S , Tamai H , Kumai M , et al. (1989) Impaired glucagon secretion to insulin-induced hypoglycemia in anorexia nervosa. Acta Endocrinol 120, 610–615. 10.1530/acta.0.1200610 2658451

[56] Kobayashi N , Tamai H , Takii M , et al. (1992) Pancreatic B-cell functioning after intravenous glucagon administration in anorexia nervosa. Acta Psychiatr Scand 85, 6–10. 1546550 10.1111/j.1600-0447.1992.tb01433.x

[57] Brown NW , Ward A , Surwit R , et al. (2003) Evidence for metabolic and endocrine abnormalities in subjects recovered from anorexia nervosa. Metabolism 52, 296–302. 12647266 10.1053/meta.2003.50067

[58] Casper RC , Pandey G , Jaspan JB , et al. (1988) Eating attitudes and glucose tolerance in anorexia nervosa patients at 8-year followup compared to control subjects. Psychiatry Res 25, 283–299. 3054985 10.1016/0165-1781(88)90099-6

[59] Ji J , Sundquist J & Sundquist K (2016) Association between anorexia nervosa and type 2 diabetes in Sweden: Etiological clue for the primary prevention of type 2 diabetes. Endocr Res 41, 310–316. 26906648 10.3109/07435800.2016.1141948

[60] Letiexhe MR , Scheen AJ & Lefebvre PJ (1997) Plasma leptin levels, insulin secretion, clearance and action on glucose metabolism in anorexia nervosa. Eat Weight Disord 2, 79–86. 14655846 10.1007/BF03339953

[61] Zuniga-Guajardo S , Garfinkel PE & Zinman B (1986) Changes in insulin sensitivity and clearance in anorexia nervosa. Metabolism 35, 1096–1100. 3537630 10.1016/0026-0495(86)90021-1

[62] Sathiaraj E , Gupta S , Chutke M , et al. (2010) Malnutrition is not an etiological factor in the development of tropical pancreatitis – a case-control study of southern Indian patients. Trop Gastroenterol 31, 169–174. 21560520

[63] Smith SR , Edgar PJ , Pozefsky T , et al. (1975) Insulin secretion and glucose tolerance in adults with protein-calorie malnutrition. Metabolism 24, 1073–1084. 807787 10.1016/0026-0495(75)90101-8

[64] Kanis JA , Brown P , Fitzpatrick K , et al. (1974) Anorexia nervosa: a clinical, psychiatric, and laboratory study. I. Clinical and laboratory investigation. Q J Med 43, 321–338. 4136528

[65] Filteau S , Praygod G , Rehman AM , et al. (2021) Prior undernutrition and insulin production several years later in Tanzanian adults. Am J Clin Nutr 113, 1600–1608. 33740034 10.1093/ajcn/nqaa438 PMC8168356

[66] Finer S , Iqbal MS , Lowe R , et al. (2016) Is famine exposure during developmental life in rural Bangladesh associated with a metabolic and epigenetic signature in young adulthood? A historical cohort study. BMJ Open 6, e011768. 10.1136/bmjopen-2016-011768 PMC5168545 27881521

[67] Hult M , Tornhammar P , Ueda P , et al. (2010) Hypertension, diabetes and overweight: looming legacies of the Biafran famine. PLOS ONE 5, e13582. 21042579 10.1371/journal.pone.0013582 PMC2962634

[68] Li Y , He Y , Qi L , et al. (2010) Exposure to the Chinese famine in early life and the risk of hyperglycemia and type 2 diabetes in adulthood. Diabetes 59, 2400–2406. 20622161 10.2337/db10-0385 PMC3279550

[69] Meng R , Lv J , Yu C , et al. (2018) Prenatal famine exposure, adulthood obesity patterns and risk of type 2 diabetes. Int J Epidemiol 47, 399–408. 29161448 10.1093/ije/dyx228 PMC5913613

[70] Portrait F , Teeuwiszen E & Deeg D (2011) Early life undernutrition and chronic diseases at older ages: the effects of the Dutch famine on cardiovascular diseases and diabetes. Soc Sci Med 73, 711–718. 21816529 10.1016/j.socscimed.2011.04.005

[71] Stanner SA , Bulmer K , Andres C , et al. (1997) Does malnutrition in utero determine diabetes and coronary heart disease in adulthood? Results from the Leningrad siege study, a cross sectional study. BMJ 315, 1342–1348. 9402775 10.1136/bmj.315.7119.1342 PMC2127836

[72] Sun Y , Zhang L , Duan W , et al. (2018) Association between famine exposure in early life and type 2 diabetes mellitus and hyperglycemia in adulthood: Results from the China Health And Retirement Longitudinal Study (CHARLS). J Diabetes 10, 724–733. 29451367 10.1111/1753-0407.12653

[73] Thurner S , Klimek P , Szell M , et al. (2013) Quantification of excess risk for diabetes for those born in times of hunger, in an entire population of a nation, across a century. Proc Natl Acad Sci USA 110, 4703–4707. 23487754 10.1073/pnas.1215626110 PMC3607051

[74] van Abeelen AF , Elias SG , Bossuyt PM , et al. (2012) Famine exposure in the young and the risk of type 2 diabetes in adulthood. Diabetes 61, 2255–2260. 22648386 10.2337/db11-1559 PMC3425424

[75] Wang N , Wang X , Han B , et al. (2015) Is exposure to famine in childhood and economic development in adulthood associated with diabetes? J Clin Endocrinol Metab 100, 4514–4523. 26509871 10.1210/jc.2015-2750 PMC4667167

[76] Wang N , Cheng J , Han B , et al. (2017) Exposure to severe famine in the prenatal or postnatal period and the development of diabetes in adulthood: an observational study. Diabetologia 60, 262–269. 27807599 10.1007/s00125-016-4148-4

[77] Wang Z , Zou Z , Yang Z , et al. (2018) The association between fetal-stage exposure to the China famine and risk of diabetes mellitus in adulthood: results from the China health and retirement longitudinal study. BMC Public Health 18, 1205. 30367620 10.1186/s12889-018-6134-x PMC6204016

[78] Wang J , Li Y , Han X , et al. (2016) Exposure to the Chinese famine in childhood increases type 2 diabetes risk in adults. J Nutr 146, 2289–2295. 27629572 10.3945/jn.116.234575

[79] Zhang Y , Liu X , Wang M , et al. (2018) Risk of hyperglycemia and diabetes after early-life famine exposure: a cross-sectional survey in Northeastern China. Int J Environ Res Public Health 15, 1125. 29857478 10.3390/ijerph15061125 PMC6024897

[80] Lu J , Li M , Xu Y , et al. (2020) Early life famine exposure, ideal cardiovascular health metrics, and risk of incident diabetes: findings from the 4C study. Diabetes Care 43, 1902–1909. 32499384 10.2337/dc19-2325

[81] Woo J , Leung JC & Wong SY (2010) Impact of childhood experience of famine on late life health. J Nutr Health Aging 14, 91–95.2012695410.1007/s12603-009-0193-8

[82] Zheng X , Wang Y , Ren W , et al. (2012) Risk of metabolic syndrome in adults exposed to the great Chinese famine during the fetal life and early childhood. Eur J Clin Nutr 66, 231–236. 21970943 10.1038/ejcn.2011.161

[83] Zhou J , Sheng J , Fan Y , et al. (2019) The effect of Chinese famine exposure in early life on dietary patterns and chronic diseases of adults. Public Health Nutr 22, 603–613. 30526705 10.1017/S1368980018003440 PMC10260494

[84] World Health Organization (2020) Severe Acute Malnutrition. Geneva: WHO.

[85] Prendergast A & Kelly P (2012) Enteropathies in the Developing World: Neglected Effects on Global Health. Am J Trop Med Hyg 86, 756–763. 22556071 10.4269/ajtmh.2012.11-0743 PMC3335677

[86] Bandsma RH , Spoelstra MN , Mari A , et al. (2011) Impaired glucose absorption in children with severe malnutrition. J Pediatr 158, 282–287.e281. 20843523 10.1016/j.jpeds.2010.07.048

[87] Rehfeld JF (2018) The origin and understanding of the incretin concept. Front Endocrinol (Lausanne) 9, 387. 30061863 10.3389/fendo.2018.00387 PMC6054964

[88] Wells JC (2009) Historical cohort studies and the early origins of disease hypothesis: making sense of the evidence. Proc Nutr Soc 68, 179–188. 19245738 10.1017/S0029665109001086

[89] Fekadu S , Yigzaw M , Alemu S , et al. (2010) Insulin-requiring diabetes in Ethiopia: associations with poverty, early undernutrition and anthropometric disproportion. Eur J Clin Nutr 64, 1192–1198. 20664624 10.1038/ejcn.2010.143

[90] Kibirige D , Lumu W , Jones AG , et al. (2019) Understanding the manifestation of diabetes in sub Saharan Africa to inform therapeutic approaches and preventive strategies: a narrative review. Clin Diabetes Endocrinol 5, 2. 30783538 10.1186/s40842-019-0077-8 PMC6376682

[91] World Health Organization (2009) WHO Child Growth Standards and the Identification of Severe Acute Malnutrition in Infants and Children. Geneva: WHO. 24809116

[92] Wellcome Trust (1970) Classification of infantile malnutrition. Lancet 2, 302–303. 4194372

