# Supplementary material for: Association of postnatal severe acute malnutrition with pancreatic exocrine and endocrine function in children and adults: a systematic review
Source: Br J Nutr. 2022 May 4;129(4):576–609. doi: 10.1017/S0007114522001404 (PMC9899575; doi:10.1017/S0007114522001404)
Supplement: Supplementary file 1 [file S0007114522001404sup.zip › S0007114522001404sup001.docx]

**Supplementary Material 1: Literature search strategy**

1. To search undernutrition/Severe Acute Malnutrition term, we used the following MeSH terms: "Malnutrition" or "Protein-Energy Malnutrition" or "Infant Nutrition Disorders" or "Severe Acute Malnutrition" or "Child Nutrition Disorders" or "Fetal Nutrition Disorders" or "Kwashiorkor" or "Starvation" or "Protein Deficiency" or "Hunger";
2. Maln* or Protein-Energy Maln* or Severe Acute Maln* or Starv* OR "Protein Deficien*
3. MeSH terms for pancreatic disorders included: "Islets of Langerhans" or "Pancreas" or "Pancreas, Exocrine" or "Exocrine Pancreatic Insufficiency" or "Cystic Fibrosis"**;**
4. Pancrea* or “Pancreatic insufficien* or "Exocrine Pancreatic Insufficien*;
5. MeSH terms for diabetes included: "Anorexia Nervosa" or “Diabetes Mellitus" or "Glucose Intolerance", or "Diabetes Complications”, or "Diabetes Insipidus, Nephrogenic" or "Diabetes, Gestational" or "Diabetes Mellitus, Type 2" or "Diabetes Mellitus, Type 1" or "Diabetes Mellitus, Experimental" or "Latent Autoimmune Diabetes in Adults" or "Polyendocrinopathies, Autoimmune" or "Hemochromatosis" or "Pregnancy in Diabetics" or "Donohue Syndrome";
6. Diabet*[tiab] or Pancreatic diabet*[tw] or Glucose Intoleranc*[tiab];
7. MeSH terms for anorexia included: "Anorexia Nervosa"
8. MeSH terms for famine included: "Famine" or "Hunger" or "Starvation" or Starv*[tiab]
9. (#1 or #2)
10. (#3 or #4)
11. (#5 or #6)
12. (#6 and #7)
13. (#9 and #6)
14. (#8 and #9)
15. (#8 and #10)
16. (#9 and #10)
17. (#10 and #11)

**PubMed Search**

((((((("Malnutrition"[Mesh] OR "Protein-Energy Malnutrition"[Mesh] OR "Severe Acute Malnutrition"[Mesh] OR "Kwashiorkor"[Mesh] OR “Starvation”[Mesh] OR "Protein Deficiency"[Mesh] OR “Hunger”[Mesh]))))))) AND (((((((("Islets of Langerhans"[Mesh] OR "Pancreas"[Mesh] OR "Pancreas, Exocrine"[Mesh] OR "Exocrine Pancreatic Insufficiency"[Mesh] OR "Cystic Fibrosis"[Mesh] OR "Glucose Intolerance"[Mesh] OR "Diabetes Mellitus"[Mesh])))))))) OR ("Famine"[text]) AND (((("Pancreas"[Mesh]) OR "Pancreas, Exocrine"[Mesh]) OR "Exocrine Pancreatic Insufficiency"[Mesh]) OR "Glucose Intolerance"[Mesh] OR "Diabetes Mellitus"[Mesh]) OR ("Famine"[Mesh]) AND (“Pancreatic insufficien*[tiab]) OR "Diabetes Mellitus"[Mesh]”) OR (((((("Maln*[tiab]" OR "Protein-Energy Maln*[tiab]" OR "Severe Acute Maln*[tiab]" OR "Kwashiorkor"[Mesh] OR “Starv*[tiab]" OR "Protein Deficien*[tiab]"OR "Hunger"[Mesh]])))))) AND ((((("Islets of Langerhans"[Mesh] OR "Pancrea*[tiab]" OR "Pancreatic, Exocrine"[Mesh] OR "Exocrine Pancreatic Insufficien*[tiab]" OR "Cystic Fibrosis"[Mesh]))))) AND (("Diabetes Mellitus"[Mesh]) OR "Glucose Intoleranc*[tiab]")) OR ((“Anorexia nervosa”[tw] AND ("Pancreatic diabet*”[tw])) OR ((“Cystic fibrosis”[Mesh]) OR “(Pancreatic insufficien*”[tw])) AND ((Diabetes) NOT “Genetic” NOT “Mice” NOT "Lung" NOT "Pulmonary" NOT "Renal" NOT "Liver disease") OR (("Interaction between exocrine and endocrine pancreatic cells") NOT "Epigenetics" NOT "Mouse" NOT "Liver" NOT "Liver-cell") OR ((("Kwashiorkor"[Mesh] OR "Protein-Energy Malnutrition"[Mesh] AND "Exocrine Pancreatic Insufficiency"[Mesh]))) OR ((((((“Kwashiorkor” [Mesh]) OR “Marasmus” [tw]) OR "Protein Energy Deficiency" [tw]) AND “Pancreatic insufficiency” [Mesh]) OR "Tropical Diabetes" [tw]) OR (("Exocrine pancreatic insufficiency" [MeSH]) AND "Diabetes") OR ((((("Malnutrition"[Mesh] OR "Protein-Energy Malnutrition"[Mesh] OR "Infant Nutrition Disorders"[Mesh] OR "Severe Acute Malnutrition"[Mesh] OR "Child Nutrition Disorders"[Mesh]))))) AND (Pancrea*[tiab]) AND (Diabet*[tiab]) OR (((("Malnutrition"[Mesh] OR "Protein-Energy Malnutrition"[Mesh] OR "Severe Acute Malnutrition"[Mesh])))) AND (("Endocrine insufficiency" OR "Exocrine insufficiency" OR "Diabetes mellitus")) OR (((“Anorexia Nervosa”[Mesh]) AND "Exocrine Pancreatic Insufficiency"[Mesh]) OR "Pancreatic Insufficiency"[tw])) OR ((“Anorexia Nervosa”[Mesh]) AND "Diabetes Mellitus"[Mesh]) OR (((("Malnutrition"[Mesh]) OR "Protein-Energy Malnutrition"[Mesh]) OR "Severe Acute Malnutrition"[Mesh])) AND (("Pancreas, Exocrine"[Mesh] OR "Cystic Fibrosis"[Mesh] OR "Islets of Langerhans"[Mesh] OR "Pancreas"[Mesh]) AND ( "Diabetes Complications"[Mesh] OR "Diabetes Mellitus"[Mesh] OR "Diabetes Mellitus, Type 2"[Mesh] OR "Diabetes Mellitus, Type 1"[Mesh] OR "Latent Autoimmune Diabetes in Adults"[Mesh] OR "Polyendocrinopathies, Autoimmune"[Mesh] OR "Hemochromatosis"[Mesh] OR "Donohue Syndrome"[Mesh])

**Searches in Google Scholar**

(“Malnutrition”[tw] AND “Etiology of pancreatic exocrine insufficiency”[tw] OR “Exocrine-endocrine pancreatic interaction, in humans”[tw] OR “Subclinical pancreatitis, in humans”[tw]) OR (“Malnutrition”[tw] AND “Tropical Chronic Pancreatitis” [tw]) OR (“Long-term” [tw] AND “malnutrition”[tw] AND Afro-Asian Pancreatitis”[tw]) OR (“Long-term malnutrition" AND "Pancreatic insufficiency" OR Diabetes in tropical countries” [tw]) OR (“Insulin secretion" AND protein-calorie malnutrition” [tw]) OR (“Anorexia nervosa”[tw] AND Pancreatic insufficiency[tw] OR Diabetes"[tw]) OR (“Malnutrition” OR cystic fibrosis AND pancreatic” OR diabetes” [tw]) OR ("Famine"[tw] AND “Pancreatic insufficiency”[tw]) OR "Diabetes Mellitus"[tw])

**Searches in Web of Science**

(“Famine” AND “Diabetes”) OR (“Long-term malnutrition” AND “Diabetes”) OR (“Undernutrition” AND “Diabetes”) OR (“Malnutrition” AND “Diabetes”) OR (“Undernutrition” AND “Diabetes” OR “Pancreatic exocrine insufficiency”) OR (“Undernutrition” AND “Diabetes” OR “Pancreatic endocrine insufficiency”) OR ("Famine"[tw] AND “Pancreatic insufficiency”[tw] OR "Diabetes Mellitus"[tw])
