# Supplementary material for: Association of postnatal severe acute malnutrition with pancreatic exocrine and endocrine function in children and adults: a systematic review
Source: Br J Nutr. 2022 May 4;129(4):576–609. doi: 10.1017/S0007114522001404 (PMC9899575; doi:10.1017/S0007114522001404)
Supplement: Supplementary file 1 [file S0007114522001404sup.zip › S0007114522001404sup003.docx]

**Supplementary Table 1: List of excluded studies with reasons from those with available full text published in English**

|  | **Author name** | **Year of publication** | **Study title** | **Reason for exclusion** |
| --- | --- | --- | --- | --- |
| 1 | Abdulkadir et al. | 1990 | The clinical and hormonal (C-peptide and glucagon) profile and liability to ketoacidosis during nutritional rehabilitation in Ethiopian patients with malnutrition-related diabetes mellitus | Not eligible; Patients were recruited because they were diabetic, not necessarily because MN |
| 2 | Abu-Bakare et al. | 1986 | Tropical or malnutrition-related diabetes- a real syndrome? | Review article |
| 3 | Adler et al. | 2007 | Characteristics of adults with and without cystic fibrosis-related diabetes mellitus | Not eligible; CF diabetic patients studied but no groups had undernourished individuals |
| 4 | Aguilar-parada et al. | 1969 | Effects of starvation on plasma pancreatic glucagon in normal man. | Not eligible; exposure was not malnutrition, starvation effect measured in healthy individuals |
| 5 | Alemu et al. | 2009 | Insulin-requiring diabetes in rural Ethiopia- should we reopen the case for malnutrition-related diabetes? | Not eligible; Does not meet study eligibility criteria |
| 6 | Anoop et al. | 2021 | Exocrine pancreatic insufficiency related fat malabsorption and its association with autonomic neuropathy in Asian Indians with type 2 diabetes mellitus | Not eligible; does not assess MN as an exposure |
| 7 | Bajaj et al. | 1987 | A Mathematical Model for Insulin Kinetics and its Application to Protein-deficient (Malnutrition-related) Diabetes Mellitus (PDDM) | Not eligible; nothing mentioned about study participants and pancreatic function measurement |
| 8 | Balcha et al. | 2018 | Type 1 Diabetes in a Resource-Poor Setting: Malnutrition Related, Malnutrition Modified, or Just Diabetes? | Review article |
| 9 | Ballmann et al. | 2014 | Open randomized prospective comparative multi-centre intervention study of patients with cystic fibrosis and early diagnosed diabetes mellitus | Study protocol |
| 10 | Bandsma et al. | 2011 | Impaired Glucose Absorption in Children with Severe Malnutrition | Not eligible; excluded because of different outcome of interest |
| 11 | Baranowska et al. | 2003 | The role of neuropeptides in the disturbed control of appetite and hormone secretion in eating disorders | Not eligible; study outcome is different, not related to pancreatic disease |
| 12 | Bartels et al. | 2018 | The Relation Between Malnutrition and the Exocrine Pancreas | Review article |
| 13 | Bawadi et al. | 2012 | Food insecurity is related to glycemic control deterioration in patients with type 2 diabetes | Not eligible; outcome food security, diabetes status was exposure |
| 14 | Bechtold et al. | 2018 | Diabetes mellitus in pediatric solid organ recipients without and with cystic fibrosis- An analysis from the German‐Austrian diabetes database | Not eligible; CF patients were not undernourished |
| 15 | Becker et al. | 1983 | The Endocrine response to Protein Calorie Malnutrition | Review article |
| 16 | Belsey et al. | 1961 | Pathological findings in the liver, pancreas and parotid gland in kwashiorkor | Short communication and result is not constrictive |
| 17 | Bercovich et al. | 2014 | Long-term health effects in adults born during the Holocaust. | Not eligible; prenatal/early life exposure to huger [born during WWII]) could not be differentiated |
| 18 | Berg et al. | 1979 | Exocrine Pancreatic Insufficiency, Small Intestinal Dysfunction and Protein Intolerance | Short communication |
| 19 | Bhatia et al. | 1995 | Exocrine pancreatic and β-cell function in malnutrition related diabetes among North Indians | Not eligible; Malnutrition not the exposure |
| 20 | Bismuth et al | 2008 | Glucose tolerance and insulin secretion, morbidity, and death in patients with cystic fibrosis | Not eligible; CF patients were not undernourished |
| 21 | Blackburn et al. | 1969 | The Pancreas in Kwashiorkor | Not eligible; an electronic microscopic study |
| 22 | Blackman et al. | 2016 | Endocrine Disorders in Cystic Fibrosis. | Not eligible; excluded because of MN was not exposure and outcome of interest different |
| 23 | Blagosklonny et al. | 2011 | Rapamycin-induced glucose intolerance- Hunger or starvation diabetes | Review article |
| 24 | Bonin-Guillaume et al. | 2006 | Insulinemia and leptinemia in geriatric patients: markers of the metabolic syndrome or of undernutrition? | Not eligible; study outcome is different, not related to pancreatic function |
| 25 | Brasel et al. | 1980 | Endocrine Adaptation to Malnutrition | Review article |
| 26 | Brooks et al. | 1993 | Ultrastructure of the islets of Langerhans in protein-energy malnutrition | Not eligible; not relevant outcome of interest |
| 27 | Brown et al. | 2014 | Anorexia Nervosa Complicated by Diabetes Mellitus- The Case for Permissive Hyperglycemia | Case report, 1 case included |
| 28 | Cao et al. | 2014 | The endocrine role between β cells and intra-islet endothelial cells | Review article |
| 29 | Carroccio et al. | 1990 | Prevalence of Diabetes Mellitus and Impaired Glucose Tolerance in Cystic Fibrosis | Short communication |
| 30 | Castillo et al. | 1985 | Insulin-stimulated glucose disposal is not increased in anorexia nervosa. | Not eligible; Focused on insulin resistance, not pancreas function |
| 31 | Chandraprasert et al. | 1976 | Diabetes Mellitus and Tropical Form of Chronic Calcific Pancreatitis in Thailand | Not eligible; N=6 juvenile diabetes, MN not assessed as exposure |
| 32 | Chari et al. | 1992 | Comparative study of the clinical profiles of alcoholic chronic pancreatitis and tropical chronic pancreatitis in Tamil Nadu, south India | Not eligible; not relevant outcome of interest |
| 33 | Chari et al. | 1990 | Faecal chymotrypsin assay in tropical and alcoholic chronic pancreatitis. | Not eligible; pancreatic function measured in chronic pancreatic patients. MN not assessed as exposure |
| 34 | Charles et al. | 1961 | Occurrence of Diabetes Mellitus in Families of Patients with Cystic Fibrosis of the Pancreas | Not eligible; nutritional status was not considered as exposure |
| 35 | Chattopadhyay et al. | 1995 | Malnutrition related diabetes mellitus (MRDM), Not Diabetes-Related Malnutrition | Short report (n=8), nothing mentioned about pancreatic function assessment |
| 36 | Chattopadhyay et al. | 1996 | Malnutrition related diabetes mellitus (MRDM) in preschool children | Letters to editor |
| 37 | Chen et al. | 2011 | The Complex Exocrine Endocrine Relationship and secondary exocrine diabetes mellitus | Review article |
| 38 | Chen et al. | 2011 | The worldwide epidemiology of type 2 diabetes mellitus--present and future perspectives. | Review article |
| 39 | Cheung et al. | 2018 | Hedonic hunger and glycemic control in non-obese and obese patients with type 2 diabetes | Not eligible; outcome was glycemic control. MN not assessed as exposure. |
| 40 | Choudhuri et al. | 2008 | Tropical calcific pancreatitis. | Review article |
| 41 | Chowdhury et al. | 2016 | Pancreatic exocrine insufficiency: Comparing fecal elastase 1 with 72-h stool for fecal fat estimation. | Not eligible; excluded because of patients recruited with chronic pancreatitis |
| 42 | Clark et al. | 1995 | Pancreatic pathology in non-insulin dependent diabetes (NIDDM) | Review article |
| 43 | Cleghorn et al. | 1991 | Exocrine pancreatic dysfunction in malnourished Australian Aboriginal children | Not eligible; not acute -MN children were included) |
| 44 | Cobelli et al. | 2017 | Exocrine and Endocrine Interactions in Cystic Fibrosis- A Potential Key to Understanding Insulin Secretion in Health and Disease? | Not eligible; Genetic study |
| 45 | Coderre et al. | 2012 | LDL-cholesterol and insulin are independently associated with body mass index in adult cystic fibrosis patients. | Not eligible; exposure was CF, not MN |
| 46 | Cohen et al. | 2005 | Fecal elastase- pancreatic status verification and influence on nutritional status in children with cystic fibrosis | Not eligible; exposure pancreatic function, not MN |
| 47 | Cox et al. | 2011 | Elizabeth Evans Hughes—surviving starvation therapy for diabetes | Journal Perspective |
| 48 | Cox et al. | 1983 | Biochem ultrasound abnormalities anorexia nervosa. | Case report |
| 49 | Crisp et al. | 1967 | Insulin response to a rapid. Intravenous injection of dextrose in patients with anorexia nervosa and obesity. | Case report |
| 50 | Crow et al. | 1998 | Eating disorders and insulin-dependent diabetes mellitus | Review article |
| 51 | Cuntz et al. | 2000 | Interrelationships between the size of the pancreas and the weight of patients with eating disorders | Not eligible; outcome not pancreatic function, only pancreatic size measured among eating disordered patients) |
| 52 | Curran et al. | 2009 | Diabetes Mellitus and Bone Disease in Cystic Fibrosis | Review article |
| 53 | Czako et al. | 2009 | Interactions between the Endocrine and Exocrine Pancreas and Their Clinical Relevance | Review article |
| 54 | Dabadghao et al. | 1996 | Islet-cell antibodies in malnutrition-related diabetes mellitus from north India | Not eligible; malnutrition is not an exposure |
| 55 | Das et al. | 2014 | Relationship between the exocrine and endocrine pancreas after acute pancreatitis | Systematic review |
| 56 | de la Iglesia et al. | 2019 | Pancreatic exocrine insufficiency and cardiovascular risk in patients with chronic pancreatitis: A prospective, longitudinal cohort study. | Not eligible; patients recruited with chronic pancreatitis |
| 57 | de Rooij et al. | 2006 | Glucose tolerance at age 58 and the decline of glucose tolerance in comparison with age 50 in people prenatally exposed to the Dutch famine | Not eligible; exposure is pre-natal MN |
| 58 | De Rooij et al | 2014 | Famines in the Last 100 Years: Implications for Diabetes. | Review article |
| 59 | Descos et al. | 1977 | Exocrine pancreatic insufficiency and primitive malnutrition | Not eligible: N=4 children studied |
| 60 | Dooley R/ Shwachman et al. | 1956 | Cystic Fibrosis of the Pancreas with Varying Degrees of Pancreatic Insufficiency | Not eligible: Case report, 4 cases included, no constructive results shown |
| 61 | Dreiling et al. |  | The pancreatic secretion in the malabsorption syndrome and related malnutrition states | Not eligible: Study outcome is not relevant (outcome was malabsorption) |
| 62 | Ejaz et al. | 2013 | Managing type 1 diabetes A journey from starvation to insulin | Not eligible (outcome was not relevant, studied type-1 diabetic patients) |
| 63 | Elder et al. | 2007 | Glucose tolerance, insulin secretion, and insulin sensitivity in children and adolescents with cystic fibrosis and no prior history of diabetes. | Not eligible; excluded because of exposure was CF, not MN |
| 64 | Ellison GTH | 2013 | The health in later life of channel Islanders exposed to the 1940-1945 occupation and siege. In. Lumey LH, Vaiserman AM, editors. Early Life Nutrition, Adult Health and Development: Lessons from Changing Dietary Patterns, Famines and Experimental Studies. Nova Science Publishers, 2013: 77-108. | Book chapter |
| 65 | Emily et al. | 2022 | Complications of chronic pancreatitis in children | Not eligible; children with chronic pancreatitis included |
| 66 | Ewald et al. | 2013 | Diabetes mellitus secondary to pancreatic diseases (Type 3c) — Are we neglecting an important disease? | Review article |
| 67 | Fedotkina et al. | 2021 | Perinatal famine is associated with excess risk of proliferative retinopathy in patients with type 2 diabetes | Not eligible; outcome not relevant, studied type-2 diabetic patients) |
| 68 | Fekadu et al. | 2010 | Insulin-requiring diabetes in Ethiopia: associations with poverty, early undernutrition and anthropometric disproportion | Not eligible; only 10 early undernourished participants; but only 8 had later diabetes |
| 69 | Fery et al. | 1990 | Mechanisms of starvation diabetes: A study with double tracer and indirect calorimetry | Not eligible; estimated starvation effect on pancreatic function among healthy individuals |
| 70 | Fink et al. | 1974 | Glucose-Induced Insulin Release Patterns: Effect of Starvation | Not eligible; Case control study, acute MN exposure not well enough defined |
| 71 | Francis et al. | 2017 | Rising trend of diabetes mellitus amongst the undernourished: State -of- the -art review. | Review article |
| 72 | Foster et al. | 2020 | Exocrine pancreatic dysfunction in type 1 diabetes | Review article |
| 73 | Galton et al. | 1971 | The effect of starvation and diabetes on glycolytic enzymes in human adipose tissue | Not eligible; different outcome of interest, not related to pancreatic function |
| 74 | Geffner et al. | 1984 | Carbohydrate tolerance in cystic fibrosis is closely linked to pancreatic exocrine function | Not eligible; different outcome of interest, MN not assessed as exposure |
| 75 | Gill et al. | 2011 | Immunological and C-peptide studies of patients with diabetes in northern Ethiopia- existence of an unusual subgroup possibly related to malnutrition | Not eligible; MN not assessed individually as exposure |
| 76 | Gillman et al. | 2015 | Prenatal famine and developmental origins of type 2 diabetes. | Not eligible; different exposure of interest (pre-natal) |
| 77 | Grey et al. | 2021 | Severe malnutrition or famine exposure in childhood and cardiometabolic non-communicable disease later in life: a systematic review | Review article |
| 78 | Hardikar et al. | 2015 | Multigenerational Undernutrition Increases Susceptibility to Obesity and Diabetes that Is Not Reversed after Dietary Recuperation | Animal study |
| 79 | Hayden et al. | 2008 | Attenuation of Endocrine‐Exocrine Pancreatic Communication in Type 2 Diabetes | Review article |
| 80 | Henry et al. | 1979 | The gastro-entero-pancreatic hormone secretion after a mixed meal in normal subjects before and after a 72-hour period of starvation | Not eligible; exposure not MN, estimated starvation effect on pancreatic function among healthy individuals |
| 81 | Herpertz et al. | 1998 | Diabetes mellitus and eating disorders- a multicenter study on the comorbidity of the two diseases | Not eligible; estimated eating disorder among diabetic patients |
| 82 | Hidayat et al. | 2019 | The influence of maternal body mass index, maternal diabetes mellitus, and maternal smoking during pregnancy on the risk of childhood-onset type 1 diabetes mellitus in the offspring: Systematic review and meta-analysis of observational studies | Review article |
| 83 | Holness et al. | 1999 | The impact of dietary protein restriction on insulin secretion and action | Not eligible; different outcome of interest |
| 84 | Holst et al. | 2020 | GIP as a Therapeutic Target in Diabetes and Obesity Insight From Incretin Co-agonists | Review article |
| 85 | Hu et al. | 2021 | Associations of early-life exposure to famine with abdominal fat accumulation are independent of family history of diabetes and physical activity. | Not eligible; excluded because of different outcome of interest |
| 86 | Humphries et al. | 1987 | Hyperamylasemia in patients with eating disorders. | Not eligible: Outcome of interest different |
| 87 | Jahoor et al. | 2006 | Glycine production in severe childhood undernutrition | Not eligible; outcome not-related to pancreatic function |
| 88 | Jialal et al. | 1987 | The occurrence of Z-type diabetes (tropical pancreatic diabetes) in the South African Indian | Case study |
| 89 | Jin et al. | 2018 | Effect of famine exposure on the risk of chronic disease in later life among population in Harbin. | Not eligible; articles not in English |
| 90 | Joffe et al. | 1963 | Pancreatic calcification in childhood associated with protein malnutrition | A case report |
| 91 | Kamala/Jaya Rao et al. | 1972 | Insulin secretion in kwashiorkor. | Review article |
| 92 | Kaye et al. | 1989 | The effect of bingeing and vomiting on hormonal secretion. | Not eligible; exposure bulimic patients, not AN |
| 93 | Keinan-Boker et al. | 2015 | Chronic health conditions in Jewish Holocaust survivors born during world war II. | Not eligible; prenatal/early life exposure to huger [born during WWII] |
| 94 | Knudsen et al. | 2015 | The development of diabetes among Danish cystic fibrosis patients over the last two decades | Not eligible; acute MN not clearly assessed as exposure. |
| 95 | Koffler et al. | 1996 | Starvation diet and very-low-calorie diets may induce insulin resistance and overt diabetes mellitus | Not eligible; Case report, 7 cases included, no constructive results shown |
| 96 | Kumar et al. | 1975 | Early Recovery of Exocrine Pancreatic Function in Adult Protein-Calorie Malnutrition | Not eligible; N=9 |
| 97 | Langley-Evans et al. | 2015 | Nutrition in early life and the programming of adult disease: a review. | Review article |
| 98 | Lanng et al. | 1944 | Insulin sensitivity and insulin clearance in cystic fibrosis patients with normal and diabetic glucose tolerance | Not eligible; CF diabetic patients studied but no groups had undernourished individuals |
| 99 | Lei et al. | 2016 | A historic study that opened a new chapter in nutritional science. | Book chapter |
| 100 | Leibovitz et al. | 2018 | Malnutrition risk is associated with hypoglycemia among general population admitted to internal medicine units. Results from the MENU study. | Not eligible; MN was outcome, not exposure |
| 101 | Lester et al. | 1993 | A search for Malnutrition-Related Diabetes Mellitus Among Ethiopian Patients | Not eligible; study outcome was nutritional status, nothing mentioned about pancreatic insufficiency |
| 102 | Ledger et al. | 2021 | Dysglycemia in Children with Severe Acute Malnutrition: A Systematic Literature Review and Meta-Analysis | Review article |
| 103 | Levine et al. | 2002 | Endocrine aspects of eating disorders in adolescents | Review article |
| 104 | Li et al. | 2017 | Chinese famine of 1959-61 and long-term health conditions | Review article |
| 105 | Li et al. | 2017 | Exposure to the Chinese famine of 1959-61 in early life and long-term health conditions: A systematic review and meta-analysis | Review article |
| 106 | Li et al. | 2017 | Prenatal exposure to famine and the development of hyperglycemia and type 2 diabetes in adulthood across consecutive generations: a population-based cohort study of families in Suihua, China. | Not eligible; different exposure of interest |
| 107 | Li et al. | 2017 | Studies into severe famine in early life and diabetes in adulthood: the need to control for differences in participant age and location. | Editorial letter |
| 108 | Li et al. | 2020 | Reply to ‘Early-life exposure to the Chinese Famine and subsequent T2DM’. | Journal correspondence |
| 109 | Li et al. | 2019 | The effect of the Chinese Famine on type 2 diabetes mellitus epidemics | Review article |
| 110 | Li et al. | 2010 | Investigation and analysis on blood glucose in famine in Chongqing. in: The 7th national academic Conference on Women and children's Nutrition of the Chinese nutrition Society and the election conference. | Abstract presented in conference |
| 111 | Li et al. | 2014 | Effects related to experiences of famine during early life on diabetes mellitus and impaired fasting glucose during adulthood | Not eligible; article not in English |
| 112 | Liu et al. | 2018 | Association of famine exposure during early life with the risk of type 2 diabetes in adulthood: a meta-analysis. | Review article |
| 113 | Liu et al. | 2019 | Effect of famine exposure during fetal period on occurrence of diabetes after adult in residents of Zhuang | Not eligible; different exposure of interest |
| 114 | Lumey et al. | 2001 | Glucose tolerance in adults after prenatal exposure to famine. | Editorial letter |
| 115 | Lumey et al. | 2015 | Association between type2 diabetes and prenatal exposure to the Ukraine famine of 1932–33: a retrospective cohort study. | Not eligible; different exposure of interest |
| 116 | Lumey et al. | 2011 | Prenatal famine and adult health. | Review article |
| 117 | Lumey et al. | 2009 | Food restriction during gestation and impaired fasting glucose or glucose tolerance and type-2 diabetes mellitus in adulthood: evidence from the Dutch Hunger Winter Families Study. | Poster presentation |
| 118 | Lundbaek et al. | 1948 | Metabolic abnormalities in starvation diabetes | Review article |
| 119 | Ma et al. | 2017 | Developmental origins of type 2 diabetes: a perspective from China. | Not eligible; different exposure of interest |
| 120 | Ma et al. | 2016 | Pancreatic exocrine insufficiency in critically ill adult patients. | Review article |
| 121 | Marks et al. | 2019 | Acute kwashiorkor in the setting of cerebral palsy and pancreatic insufficiency. | Editorial letter |
| 122 | Martins et al. | 2008 | Children recovered from malnutrition exhibit normal insulin production and sensitivity | Not eligible; Malnutrition not assessed as exposure |
| 123 | McEniry et al. | 2013 | Early-life conditions and older adult health in low- and middle-income countries: a review. | Review article |
| 124 | Megias et al. | 2015 | Influence of macrolides, nutritional support and respiratory therapies in diabetes and normal glucose tolerance in cystic fibrosis | Not eligible; acute MN not assessed as exposure |
| 125 | Middleton et al. | 2014 | Cystic Fibrosis Related Diabetes-Potential pitfalls in the transition from pediatric to adult care | Review article |
| 126 | Milner et al. | 1972 | Insulin secretion in human protein-calorie deficiency | Review article |
| 127 | Mirghani et al. | 2019 | The association between hypoglycemia and hospital use, food insufficiency, and unstable housing conditions: a cross-sectional study among patients with type 2 diabetes in Sudan. | Not eligible; exposure status was DM not MN |
| 128 | Mittal et al. | 2002 | The clinical spectrum of fibrocalculous pancreatic diabetes in north India | Not eligible; acute MN not assessed as exposure |
| 129 | Mohan et al. | 1998 | Fibrocalculous pancreatic diabetes | Review article |
| 130 | Mohan et al. | 1985 | Tropical pancreatic diabetes in South India: heterogeneity in clinical and biochemical profile | Not eligible; acute MN not assessed as exposure |
| 131 | Mohan et al. | 1983 | Pancreatic β-cell function in tropical pancreatic diabetes | Not eligible; patients recruited on the basis of diabetes, not MN (not Cases control study) |
| 132 | Mohan et al. | 1985 | Ultrasonography evaluation of the pancreas in tropical diabetes | Not eligible; different outcome of interest |
| 133 | Mohan et al. | 2005 | Natural history of endocrine failure in tropical chronic pancreatitis | Not eligible; different outcome of interest |
| 134 | Mohan et al. | 1987 | Malnutrition related diabetes mellitus | Editorial letter |
| 135 | Mohan et al. | 1989 | Exocrine pancreatic function in tropical fibrocalculous pancreatic diabetes | Not eligible; acute MN not assessed as exposure |
| 136 | Montalegre et al. | 1987 | Pancreatic Lesions Due to Prolonged Malnutrition in IBO Children | Short communication |
| 137 | Moreau et al. | 2008 | Continuous glucose monitoring in cystic fibrosis patients according to the glucose tolerance | Not eligible; MN not exposure, studied Cystic fibrosis patients |
| 138 | Mu et al. | 2008 | Gender difference in the long-term impact of famine | Report |
| 139 | Nerengerg et al. | 1953 | Regranulation of beta cells of islets of Langerhans following insulin and starvation | Animal study |
| 140 | Nettles et al. | 1990 | Diabetes secondary to cystic fibrosis- an increasing clinical problem | Review article |
| 141 | Nigatu et al. | 2012 | Epidemiology, complications and management of diabetes in Ethiopia: a systematic review. | Review article |
| 142 | Nousia-Arvanitakis et al. | 2001 | Insulin improves clinical status of patients with cystic-fibrosis-related diabetes mellitus | Not eligible; CF diabetic patients studied but no undernourished individuals |
| 143 | Nousia-Arvanitakis et al. | 1985 | Pancreatic polypeptide in cystic fibrosis | Not eligible; pancreatic function measured in normal cystic fibrosis patients, different outcome of interest |
| 144 | Nyirjesy et al. | 2018 | β-Cell secretory defects are present in pancreatic insufficient cystic fibrosis with 1-hour oral glucose tolerance test glucose ≥155 mg/dL | Not eligible; pancreatic function measured in normal cystic fibrosis patients |
| 145 | O’Riordan et al. | 2010 | Cystic fibrosis-related diabetes in childhood | Review article |
| 146 | Okuno et al. | 1990 | Prevalence and clinical features of diabetes mellitus secondary to chronic pancreatitis in Japan; a study by Questionnaire | Not eligible; different outcome of interest, nothing mentioned about pancreatic function test |
| 147 | Olurin et al. | 1969 | Pancreatic calcification- a report of 45 cases | Case study |
| 148 | Owen et al. | 1974 | Comparative measurements of glucose, beta-hydroxybutyrate, acetoacetate, and insulin in blood and cerebrospinal fluid during starvation. | Not eligible; different outcome of interest, obese subject studied |
| 149 | Papoz et al. | 1998 | Clinical classification of diabetes in tropical west Africa | Not meet study eligibility criteria |
| 150 | Peng et al. | 2021 | Vitamin D Status and Risk of Cystic Fibrosis-Related Diabetes: A Retrospective Single Center Cohort Study | Not eligible; different outcome of interest |
| 151 | Peraldo et al. | 1998 | Evaluation of glucose tolerance and insulin secretion in cystic fibrosis patients | Not eligible; MN was likely secondary to cystic fibrosis |
| 152 | Perito et al. | 2022 | Complications of chronic pancreatitis in children | Not eligible; children with chronic pancreatitis included |
| 153 | Petersen et al. | 2002 | Tropical Pancreatitis | Clinical Review |
| 154 | Pimstone et al. | 1976 | Endocrine function in protein-calorie malnutrition | Review article |
| 155 | Pitchumoni et al. | 1973 | Pancreas in primary malnutrition disorders | Review article |
| 156 | Pitchumoni et al. | 1973 | Chronic Cassava Toxicity: Possible relationship to chronic pancreatic disease in malnourished populations | Letters to editor |
| 157 | Potter et al. | 2021 | Combined Indeterminate and Impaired Glucose Tolerance Is a Novel Group at High Risk of Cystic Fibrosis-Related Diabetes | Not eligible; study outcome is different |
| 158 | Portha et al. | 2011 | Early-life origins of type 2 diabetes: fetal programming of the beta-cell mass. | Animal study |
| 159 | Powers et al. | 1983 | Anorexia Nervosa and Diabetes Mellitus | Case report, 4 cases included, no constructive results shown |
| 160 | Qin et al. | 2021 | Undernutrition when young and the risk of poor renal function in adulthood in women with diabetes in Shanghai, China | Not eligible; study outcome is different, not related to pancreatic disease |
| 161 | Rafii et al. | 2005 | Changes in response to insulin and the effects of varying glucose tolerance on whole-body protein metabolism in patients with cystic fibrosis | Not eligible; study outcome is different, not related to pancreatic disease) |
| 162 | Rajasurlya et al. | 1970 | Pancreatic Calcification Following Prolonged Malnutrition | A case report |
| 163 | Ramachandran et al. | 1988 | Clinical features of diabetes in the young as seen at a diabetes centre in South India | Not eligible; outcome is clinical features and nutritional status |
| 164 | Rao et al. | 1993 | Is Tropical Pancreatic Diabetes Malnutrition Related? | Review article |
| 165 | Rao et al. | 1984 | The Role of Undernutrition in the Pathogenesis of Diabetes Mellitus | Review article |
| 166 | Rao et al. | 1990 | CHRONIC UNDERNUTRITION MAY ACCENTUATE THE BETA-CELL DYSFUNCTION OF TYPE-2 DIABETES | Not eligible; exposure chronic MN |
| 167 | Rashak et al | 2019 | Diabetes, undernutrition, migration and indigenous communities: tuberculosis in Chiapas, Mexico. | Not eligible; exposure and outcome of interest different |
| 168 | Ratnaike et al. | 1963 | Pancreatic Calcification Related to Protein Malnutrition | A Case report, 1 case included |
| 169 | Ravelli et al. | 1998 | Glucose tolerance in adults after prenatal exposure to famine | Not eligible; only pre-natal MN included |
| 170 | Reusens et al. | 2011 | Maternal malnutrition programs the endocrine pancreas in progeny. | Animal study |
| 171 | Robertson et al. | 1990 | Insulin-Dependent Diabetes Mellitus | Not eligible; exposure IDDM |
| 172 | Rolfe et al. | 1987 | Tropical pancreatic diabetes in Zambia. | Not eligible; missing age and/or age groups information. outcome only assessed pancreatic calcification status. |
| 173 | Roseboom et al. | 2011 | Hungry in the womb: what are the consequences? Lessons from the Dutch famine. | Review article |
| 174 | Roseboom et al. | 2006 | The Dutch famine and its long-term consequences for adult health. | Review article |
| 175 | Saraya et al. | 1999 | Is protein-deficient diabetes mellitus a pancreatitis? | Not eligible: acute MN not assessed as exposure. |
| 176 | Saraya et al. | 2003 | A pancreaticographic study of malnutrition-related diabetes mellitus. | Not eligible; different outcome of interest |
| 177 | Sarles et al. | 1994 | Pancreatic Lesions And Modification of Pancreatic Juice | Not eligible; different outcome of interest |
| 178 | Sawaya et al. | 2009 | Malnutrition, long-term health and the effect of nutritional recovery | Review article |
| 179 | Schwachman et al. | 1952 | Nutritional Disturbances in Childhood, with Particular Emphasis on the Celiac Syndrome and Pancreatic Insufficiency | Seminar lecture |
| 180 | Seligman et al. | 2010 | Hunger and socioeconomic disparities in chronic disease | Not eligible; different outcome of interest |
| 181 | Shamberger et al. | 1994 | Long-term nutritional and metabolic consequences of pancreaticoduodenectomy in children | Not eligible; MN not exposure |
| 182 | Shaper et al. | 1960 | Chronic Pancreatic Disease and Protein Malnutrition | Not eligible; different outcome of interest |
| 183 | Shi et al. | 2020 | Early life exposure to 1959-1961 Chinese famine exacerbates association between diabetes and cardiovascular disease | Not eligible; different outcome of interest |
| 184 | Shivaprasad et al. | 2015 | Pancreatic exocrine insufficiency in type 1 and type 2 diabetics of Indian origin | Not eligible; different outcome of interest |
| 185 | Silverman et al. | 1977 | Anorexia nervosa: clinical and metabolic observations in a successful treatment plan. In: VIGERSKY RA, ed. Anorexia nervosa. | Book chapter |
| 186 | Singhal et al. | 2003 | Low nutrient intake and early growth for later insulin resistance in adolescents | Not eligible; different outcome of interest |
| 187 | Smith et al. | 1975 | Insulin secretion and glucose tolerance in adults with protein-calorie malnutrition | Not eligible; different outcome of interest |
| 188 | Stephan et al. | 1982 | Correlations between plasma insulin and body weight in obesity, anorexia nervosa and diabetes mellitus | Not eligible; assessed insulin response across BMI categories |
| 189 | Sterescu et al. | 2010 | Natural History of Glucose Intolerance in Patients with Cystic Fibrosis: Ten-Year Prospective Observation Program | Not eligible; CF patients studied but not undernourished |
| 190 | Subhash et al. | 2012 | Tropical calcific pancreatitis | A case report |
| 191 | SWi et al. | 1992 | IS DIABETES-MELLITUS RELATED TO UNDERNUTRITION IN RURAL TANZANIA | Not eligible; study participants had no prior or current malnutrition exposure |
| 192 | Swislocki et al. | 2005 | Secretin−stimulated Amylase in Diabetes Mellitus | Not eligible; not used malnutrition as exposure) |
| 193 | Takii et al. | 2011 | The relationship between the age of onset of type 1 diabetes and the subsequent development of a severe eating disorder by female patients | Not eligible; eating disorder identified among diabetic patients |
| 194 | Tayek et al. | 1997 | Insulin secretion, glucose production, and insulin sensitivity in underweight and normal-weight volunteers, and in underweight and normal- weight cancer patients: A clinical research center study | Not eligible; chronic diseases after cancer cachexia |
| 195 | Taylor-Cousar et al. | 2016 | Continuous Glucose Monitoring Identifies Adult Patients at Risk for Development of Cystic Fibrosis Related Diabetes | Not eligible; only determined diabetic prevalence, not even showed association between nutritional status and diabetic outcomes) |
| 196 | Terliesner et al. | 2017 | Cystic-fibrosis related-diabetes (CFRD) is preceded by and associated with growth failure and deteriorating lung function | Not eligible; study outcome is different, not related to pancreatic disease |
| 197 | Tu et al. | 2017 | Endocrine and exocrine pancreatic insufficiency after acute pancreatitis | Not eligible; MN not exposure and pancreatic function measured in acute pancreatitis patients) |
| 198 | Tzagournis et al. | 1970 | Glucose Intolerance Mechanism after Starvation | Not eligible; discussed about 7 obese nondiabetic subject studied |
| 199 | Unger et al. | 1974 | Alpha- and Beta-cell Interrelationships in Health and Disease | Review article |
| 200 | Vaiserman et al. | 2017 | Early-Life Nutritional Programming of Type 2 Diabetes: Experimental and Quasi-Experimental Evidence. | Not eligible; different outcome of interest |
| 201 | Vaiserman et al. | 2010 | Predisposition to type II diabetes in Ukraine residents exposed to famine 1932-1933 during prenatal development. | Book chapter |
| 202 | Vannasaeng et al. | 1986 | C-peptide secretion in calcific tropical pancreatic diabetes | Not meet study eligibility criteria |
| 203 | Vujasinovic et al. | 2014 | Pancreatic exocrine insufficiency, diabetes mellitus and serum nutritional markers after acute pancreatitis | Not meet study eligibility criteria |
| 204 | Wang et al. | 1998 | In vitro influences between pancreatic adenocarcinoma cells and pancreatic islets | Animal study |
| 205 | Wang et al. | 2018 | The association of famine exposure during fetal life and gene with adulthood glucose metabolism. | Not eligible; Famine exposed during fetal period |
| 206 | Wang et al. | 2021 | Metabolites in the association between early-life famine exposure and type 2 diabetes in adulthood over a 5-year follow-up period | Not eligible; Famine exposed during fetal period |
| 207 | Weisman et al. | 2018 | Evolving trends in the epidemiology, risk factors, and prevention of type 2 diabetes: a review. | Review article |
| 208 | Winter et al. | 2004 | Impaired gastric acid and pancreatic enzyme secretion in patients with Crohn's disease may be a consequence of a poor nutritional state | Not eligible; MN secondary to Crohn’s disease |
| 209 | Wiyono et al. | 1988 | Exocrine and endocrine pancreatic function in malnutrition-related diabetes mellitus (MRDM) in Yogyakarta, Indonesia. | Recruited on the basis of diabetes |
| 210 | Wu et al. | 2020 | Risk factors for development of diabetes mellitus (Type 3C) after partial pancreatectomy: A systematic review | Review article |
| 211 | Yajnik et al. | 1993 | Fibrocalculous Pancreatic Diabetes in Pune India | Not eligible; different outcome of interest |
| 212 | Yajnik et al. | 2004 | Early life origins of insulin resistance and type 2 diabetes in India and other Asian countries | Review article |
| 213 | Ziai et al. | 2012 | The association between leptin and insulin levels in adults with cystic fibrosis | Not eligible; different outcome of interest, cystic fibrosis patients were not undernourished. |
| 214 | Zhao et al. | 2013 | Exposure to the 1959–1961 Chinese famine in early life and the risk of chronic metabolic diseases in adulthood. | Not eligible; conference proceeding |
| 215 | Zhang et al. | 2010 | Investigation on prevalence of diabetes in adults born during famine years. in: Proceedings of the twelfth national conference on behavioral medicine. | Not eligible; conference proceeding |
| 216 | Zhang et al. | 2018 | Risk of Hyperglycemia and Diabetes after Early-Life Famine Exposure: A Cross-Sectional Survey in Northeastern China | Not eligible; fetal exposure |
| 217 | Zhang et al. | 2020 | Exposure to Chinese Famine in Fetal Life and the Risk of Dysglycemia in Adulthood | Not eligible; fetal exposure |
| 218 | Zoppi et al. | 1983 | Feeding Induced Metabolic and Endocrine Responses | Review article |
| 219 | Zoppi et al. | 1968 | Protein content and pancreatic enzyme activities of duodenal juice in normal children and in children with exocrine pancreatic insufficiency | Not eligible; CF patients studied but not undernourished |
| 220 | Zou et al. | 2020 | Early-life exposure to the Chinese Famine and subsequent T2DM. | Journal correspondence |
| 221 | Zuidema et al. | 1955 | Calcification and cirrhosis of the pancreas in patients with deficient nutrition. | Not eligible; sample size <10 |
