# Supplementary material for: Association of postnatal severe acute malnutrition with pancreatic exocrine and endocrine function in children and adults: a systematic review
Source: Br J Nutr. 2022 May 4;129(4):576–609. doi: 10.1017/S0007114522001404 (PMC9899575; doi:10.1017/S0007114522001404)
Supplement: Supplementary file 1 [file S0007114522001404sup.zip › S0007114522001404sup002.doc]

**Supplementary table 3.1 Quality assessment tools for the observational study designs to assess quality/strength of the evidence.**

|  | **Assessment tools** |
| --- | --- |
| Q-1 | Was the research question or objective clearly stated? [score 0=not reported/no, 1=partially stated, 2=yes] |
| Q-2 | Was the study design clearly stated? [score 0=not reported/no, 1=partially stated, 2=yes] |
| Q-3 | Was the study population clearly defined? [score 0=not reported/no, 1=partially stated, 2=yes] |
| Q-4 | Was the sampling method/s clearly defined and appropriate for the question being asked? [score 0=no/not reported, 1=partially defined, 2=yes] |
| Q-5 | Were inclusion and exclusion criteria prespecified and applied uniformly to all participants? [score 0=no/not reported, 1=partially stated/applied, 2= yes] |
| Q-6 | Was the exposure clearly defined and implemented (malnutrition, or exposure to famine)? [score 0=no/not reported/not defined, 1=partially defined/mentioned, 2= yes] |
| Q-7 | Was there an appropriate control/comparison group? [score 0=unlikely 1=possibly, 2=probable] |
| Q-8 | Was the outcome clearly defined and implemented? [score 0=no/not reported/not defined, 1=partially defined/mentioned, 2= yes] |
| Q-9 | How reliable were the methods used for the determination of outcome (pancreatic functions)? [score 0 = poor, 1=ok, 2=good] |
| Q-10 | Was sample size justified - , power description, or variance and effect estimates provided? [score 0 = poor, 1=ok, 2=good] |
| Q-11 | Was the sample size big enough for main comparison or association being tested (or the one we are interested in for this paper if not the main objective of the paper)? [score 0=unlikely, 1=possibly, 2= yes] |
| Q-12 | If a cohort study- was the outcome of interest measured and excluded from individuals at baseline? [score 0=unlikely, 1=possibly, 2= yes] |
| Q-13 | If a cohort study - was the follow-up period sufficient to reasonably expect to see an association between exposure and outcome, if it existed? [score 0=unlikely, 1=possibly, 2= yes] |
| Q-14 | Did the study examine any dose response effect? [score 0=unlikely, 1=partially, 2= yes] |
| Q-15 | Was the duration of exposure assessed? [score 0=no/not reported, 1=partially assessed, 2= yes] |
| Q-16 | Were the outcome assessors blinded to the exposure status of participants? [score 0=unlikely, 1=possibly, 2= yes] |
| Q-17 | If a cohort study was loss to follow-up after baseline 20% or less? [score 0=no/not reported/not defined, 1=possibly, 2= yes] |
| Q-18 | Was the method of analysis appropriate? [score 0=no/not reported, 1=possibly, 2= yes] |
| Q-19 | Were key potential confounding variables measured and adjusted statistically for their impact on the relationship between exposure(s) and outcome(s)? [score 0=unlikely, 1=partially, 2= yes] |
| CD, cannot be determined (if CD, point 0)., NA, not applicable (not account in final scoring), NR, not reported (if NR, point 0)., N, no (if N/unlikely, point 0), PA, partially/possibly answered (if PA, point 1), Y, yes (if Y, point 2). ≥ 80% = High; 60-79% = Medium; , 45-59% = Low; <45% = V. Low | |

**Supplementary table 3.1 Quality assessment score for the observational study designs to assess quality/strength of the evidence.**

|  | **Q-1** | **Q-2** | **Q-3** | **Q-4** | **Q-5** | **Q-6** | **Q-7** | **Q-8** | **Q-9** | **Q-10** | **Q-11** | **Q-12** | **Q-13** | **Q-14** | **Q-15** | **Q-16** | **Q-17** | **Q-18** | **Q-19** | **Rating** |
| --- | --- | --- | --- | --- | --- | --- | --- | --- | --- | --- | --- | --- | --- | --- | --- | --- | --- | --- | --- | --- |
| **Adegbenro, 1991** | 2 | 0 | 2 | 0 | 2 | 2 | 2 | 1 | 2 | 0 | 0 | NA | NA | 0 | 0 | 1 | NA | 2 | 1 | **53.1** |
| **Barbezat, 1968** | 1 | 1 | 1 | 0 | 1 | 1 | 1 | 2 | 2 | 0 | 0 | 0 | 1 | 0 | 2 | 0 | 0 | 2 | 0 | **39.5** |
| **Bartels, 2016** | 2 | 1 | 2 | 2 | 1 | 2 | 0 | 2 | 2 | 2 | 1 | 0 | 0 | 0 | 0 | 0 | 1 | 2 | 0 | **52.6** |
| **Becker, 1971** | 1 | 1 | 2 | 0 | 2 | 2 | 1 | 2 | 2 | 0 | 0 | 0 | 1 | 1 | 1 | 1 | 0 | 1 | 0 | **47.4** |
| **Becker, 1972** | 1 | 1 | 2 | 0 | 2 | 2 | 1 | 2 | 2 | 0 | 0 | 0 | 0 | 2 | 0 | 1 | 0 | 1 | 0 | **44.7** |
| **Becker, 1975** | 1 | 1 | 1 | 0 | 2 | 2 | 0 | 2 | 2 | 0 | 0 | 0 | 1 | 2 | 0 | 1 | 0 | 2 | 0 | **44.7** |
| **Blickle, 1984** | 1 | 0 | 1 | 0 | 1 | 1 | 2 | 2 | 2 | 0 | 0 | NA | NA | 0 | 0 | 0 | NA | 1 | 0 | **34.4** |
| **Bowie, 1964** | 0 | 0 | 1 | 0 | 1 | 1 | 2 | 2 | 1 | 0 | 2 | NA | NA | 0 | 0 | 0 | NA | 0 | 0 | **31.3** |
| **Briars, 1998** | 2 | 2 | 2 | 0 | 1 | 2 | 0 | 1 | 2 | 0 | 1 | NA | NA | 1 | 0 | 0 | NA | 1 | 1 | **50.0** |
| **Brown, 2003** | 2 | 1 | 1 | 0 | 2 | 2 | 2 | 2 | 2 | 0 | 0 | NA | NA | 0 | 0 | 0 | NA | 1 | 0 | **46.9** |
| **Casper, 1988** | 2 | 1 | 2 | 0 | 2 | 2 | 2 | 2 | 2 | 0 | 0 | NA | NA | 2 | 2 | 0 | NA | 2 | 2 | **71.9** |
| **Cook, 1967** | 2 | 1 | 1 | 0 | 1 | 1 | 2 | 2 | 2 | 0 | 0 | NA | NA | 0 | 2 | 0 | NA | 0 | 0 | **43.8** |
| **Danus, 1970** | 1 | 1 | 2 | 0 | 1 | 2 | 2 | 2 | 2 | 0 | 0 | 0 | 0 | 0 | 0 | 0 | 0 | 0 | 0 | **34.2** |
| **Durie, 1985** | 2 | 0 | 1 | 0 | 0 | 2 | 2 | 2 | 2 | 0 | 0 | 0 | 0 | 1 | 0 | 1 | 1 | 1 | 0 | **39.5** |
| **El-Hodhod, 2005** | 1 | 1 | 2 | 0 | 1 | 2 | 2 | 2 | 2 | 0 | 0 | 0 | 1 | 0 | 0 | 1 | 0 | 2 | 0 | **44.7** |
| **Filtaue, 2021** | 2 | 2 | 1 | 1 | 1 | 2 | 2 | 2 | 2 | 0 | 1 | 1 | 2 | 2 | 1 | 1 | 0 | 2 | 2 | **67.5** |
| **Finer, 2016** | 2 | 2 | 2 | 1 | 1 | 2 | 2 | 2 | 2 | 0 | 0 | NA | NA | 1 | 2 | 1 | NA | 1 | 1 | **68.8** |
| **Fujii, 1989** | 2 | 1 | 2 | 0 | 2 | 2 | 2 | 2 | 2 | 0 | 1 | 0 | 1 | 0 | 2 | 0 | 2 | 2 | 0 | **60.5** |
| **Garg, 1989** | 2 | 0 | 2 | 0 | 1 | 2 | 2 | 2 | 2 | 0 | 0 | NA | NA | 2 | 0 | 1 | NA | 1 | 0 | **53.1** |
| **Hadden, 1967** | 1 | 1 | 2 | 0 | 1 | 1 | 0 | 2 | 2 | 0 | 0 | 0 | 0 | 0 | 0 | 1 | 0 | 0 | 0 | **28.9** |
| **Hult, 2010** | 2 | 2 | 2 | 2 | 2 | 2 | 2 | 2 | 2 | 0 | 1 | NA | NA | 2 | 1 | 0 | NA | 2 | 2 | **81.3** |
| **James, 1970** | 1 | 1 | 1 | 0 | 1 | 2 | 1 | 2 | 2 | 0 | 0 | NA | NA | 0 | 0 | 1 | NA | 0 | 0 | **37.5** |
| **Ji, 2016** | 2 | 2 | 2 | 2 | 2 | 2 | 2 | 2 | 2 | 0 | 2 | 2 | 2 | 2 | 0 | 0 | 1 | 2 | 2 | **81.6** |
| **Kajubi, 1972** | 2 | 1 | 2 | 0 | 1 | 2 | 1 | 2 | 2 | 0 | 0 | NA | NA | 0 | 2 | 1 | NA | 1 | 0 | **53.1** |
| **Kanis, 1974** | 2 | 1 | 2 | 0 | 2 | 2 | 0 | 2 | 2 | 0 | 0 | 0 | 1 | 0 | 1 | 0 | 0 | 0 | 0 | **39.5** |
| **Keni, 1995** | 1 | 0 | 1 | 0 | 2 | 1 | 1 | 2 | 2 | 0 | 0 | NA | NA | 0 | 0 | 1 | NA | 1 | 0 | **37.5** |
| **Kobayash, 1992** | 2 | 0 | 0 | 0 | 2 | 2 | 1 | 2 | 2 | 0 | 0 | 0 | 1 | 1 | 0 | 0 | 0 | 1 | 0 | **36.8** |
| **Kumai, 1988** | 2 | 1 | 1 | 0 | 2 | 2 | 2 | 2 | 2 | 0 | 1 | 1 | 1 | 1 | 0 | 0 | 2 | 2 | 0 | **57.9** |
| **Li, 2010** | 2 | 2 | 2 | 2 | 2 | 2 | 2 | 2 | 1 | 0 | 2 | NA | NA | 2 | 2 | 0 | NA | 2 | 2 | **84.4** |
| **Letiexhe, 1997** | 2 | 0 | 2 | 0 | 2 | 2 | 2 | 2 | 2 | 0 | 0 | NA | NA | 0 | 2 | 0 | NA | 1 | 0 | **53.1** |
| **Lu, 2020** | 2 | 2 | 2 | 1 | 2 | 2 | 2 | 2 | 2 | 0 | 1 | 2 | 1 | 2 | 2 | 1 | 0 | 2 | 2 | **78.9** |
| **Martinez Olmos, 2013** | 2 | 1 | 2 | 0 | 2 | 2 | 0 | 2 | 2 | 0 | 1 | 0 | 1 | 0 | 0 | 0 | 2 | 2 | 0 | **34.2** |
| **Meng, 2017** | 2 | 2 | 2 | 1 | 2 | 2 | 2 | 2 | 2 | 0 | 2 | 2 | 2 | 2 | 2 | 0 | 1 | 2 | 2 | **84.2** |
| **Milne, 1971** | 2 | 1 | 1 | 0 | 1 | 2 | 0 | 2 | 2 | 0 | 0 | 0 | 1 | 1 | 0 | 1 | 2 | 1 | 0 | **44.7** |
| **Pereyra , 2021** | 2 | 2 | 2 | 2 | 2 | 2 | 1 | 2 | 2 | 0 | 1 | 0 | 1 | 0 | 0 | 0 | 0 | 2 | 2 | **60.5** |
| **Portrait, 2011** | 2 | 2 | 2 | 2 | 2 | 2 | 2 | 2 | 1 | 0 | 1 | 0 | 2 | 2 | 2 | 0 | 2 | 2 | 2 | **78.9** |
| **Prinsloo, 1971** | 2 | 1 | 1 | 0 | 1 | 1 | 1 | 2 | 2 | 0 | 0 | 0 | 1 | 0 | 0 | 1 | 0 | 2 | 0 | **39.5** |
| **Sauniere, 1986** | 2 | 1 | 2 | 0 | 1 | 1 | 2 | 2 | 2 | 0 | 1 | NA | NA | 0 | 1 | 1 | NA | 2 | 0 | **56.3** |
| **Sizonenko, 1975** | 2 | 0 | 2 | 0 | 0 | 1 | 1 | 2 | 2 | 0 | 0 | NA | NA | 0 | 0 | 0 | NA | 1 | 0 | **34.4** |
| **Slone, 1961** | 2 | 0 | 1 | 0 | 1 | 2 | 2 | 2 | 2 | 0 | 0 | NA | NA | 0 | 0 | 0 | NA | 0 | 0 | **37.5** |
| **Smith, 1975** | 2 | 1 | 2 | 0 | 1 | 0 | 0 | 0 | 2 | 0 | 0 | 0 | 1 | 0 | 0 | 0 | 0 | 1 | 0 | **26.3** |
| **Spoelstra, 2012** | 2 | 1 | 1 | 0 | 2 | 2 | 1 | 2 | 2 | 2 | 2 | NA | NA | 0 | 0 | 1 | NA | 2 | 0 | **62.5** |
| **Stanner, 1997** | 2 | 2 | 2 | 1 | 2 | 2 | 2 | 2 | 2 | 2 | 1 | NA | NA | 0 | 2 | 0 | NA | 1 | 1 | **75.0** |
| **Sun, 2018** | 2 | 2 | 2 | 2 | 2 | 2 | 2 | 2 | 2 | 0 | 2 | NA | NA | 2 | 2 | 0 | NA | 2 | 2 | **87.5** |
| **Tandon, 1969** | 2 | 0 | 1 | 0 | 1 | 1 | 2 | 2 | 2 | 0 | 0 | NA | NA | 0 | 0 | 0 | NA | 0 | 0 | **34.4** |
| **Tandon, 1970** | 2 | 0 | 1 | 0 | 0 | 0 | 2 | 1 | 2 | 0 | 0 | 0 | 2 | 0 | 0 | 0 | 2 | 1 | 0 | **28.9** |
| **Thurner, 2013** | 2 | 1 | 2 | 2 | 2 | 2 | 2 | 2 | 2 | 0 | 2 | 1 | 2 | 2 | 2 | 0 | 2 | 2 | 2 | **84.2** |
| **Van Abeelen, 2012** | 2 | 2 | 2 | 2 | 2 | 2 | 2 | 2 | 1 | 0 | 1 | 2 | 2 | 2 | 2 | 0 | 1 | 2 | 2 | **81.6** |
| **Wallensteen, 1988** | 2 | 0 | 2 | 0 | 1 | 2 | 2 | 2 | 1 | 1 | 0 | NA | NA | 0 | 2 | 0 | NA | 2 | 0 | **53.1** |
| **Wang, 2015** | 2 | 2 | 2 | 2 | 2 | 2 | 2 | 2 | 2 | 0 | 2 | NA | NA | 2 | 2 | 0 | NA | 2 | 2 | **87.5** |
| **Wang, 2016** | 2 | 2 | 2 | 2 | 2 | 2 | 2 | 2 | 1 | 0 | 2 | 2 | 2 | 2 | 2 | 0 | 2 | 2 | 2 | **86.8** |
| **Wang, 2017** | 2 | 2 | 2 | 1 | 2 | 2 | 2 | 2 | 1 | 0 | 2 | NA | NA | 2 | 2 | 0 | NA | 2 | 2 | **81.3** |
| **Wang, 2018** | 2 | 1 | 2 | 1 | 2 | 2 | 2 | 2 | 2 | 0 | 1 | NA | NA | 2 | 2 | 0 | NA | 2 | 2 | **78.1** |
| **Widodo, 2016** | 2 | 2 | 2 | 1 | 2 | 0 | 2 | 1 | 2 | 0 | 1 | NA | NA | 0 | 0 | 1 | NA | 2 | 0 | **56.3** |
| **Woo, 2010** | 2 | 2 | 2 | 1 | 1 | 1 | 0 | 1 | 1 | 0 | 1 | NA | NA | 0 | 1 | 0 | NA | 1 | 1 | **46.8** |
| **Zhang, 2018** | 2 | 1 | 2 | 1 | 1 | 2 | 2 | 2 | 1 | 0 | 2 | NA | NA | 2 | 2 | 0 | NA | 2 | 2 | **75.0** |
| **Zheng, 2012** | 2 | 1 | 2 | 2 | 2 | 1 | 2 | 2 | 2 | 0 | 2 | NA | NA | 0 | 0 | 0 | NA | 2 | 0 | **62.5** |
| **Zuniga-Guajardo, 1986** | 2 | 0 | 2 | 0 | 2 | 2 | 2 | 2 | 2 | 0 | 0 | 0 | 0 | 0 | 0 | 0 | 0 | 1 | 0 | **39.5** |
| **Zhou, 2017** | 2 | 2 | 2 | 1 | 2 | 2 | 2 | 2 | 2 | 0 | 1 | NA | NA | 0 | 0 | 0 | NA | 1 | 2 | **65.6** |

**Supplementary table 3.2 Quality assessment tools for the intervention/randomized control trial study designs to assess quality/strength of the evidence.**

|  | **Assessment tools** |
| --- | --- |
| Q-1 | Was the research question or objective clearly stated? [score 0=not reported/no, 1=partially stated, 2=yes] |
| Q-2 | Was there a comparison arm? If Yes was treatment randomised? [score 0=no comparison, 1=non-randomised , 2= randomised] |
| Q-3 | Was the study design clearly stated? [score 0=not reported/no, 1=partially stated, 2=yes] |
| Q-4 | Was the study population clearly defined? [score 0=not reported/no, 1=partially stated, 2=yes] |
| Q-5 | Was the sampling method/s clearly defined and appropriate for the question being asked? [score 0=no/not reported, 1=partially defined, 2=yes] |
| Q-6 | If RCT– was the method of randomization appropriate (use of randomly generated assignment)? [score 0=no/not reported, 1=partially performed, 2= randomised appropiately] |
| Q-7 | If RCT was there concealment of treatment allocation? [score 0=no/not reported, 1=partially defined, 2=yes] |
| Q-8 | If NOT a single arm trial – were the groups similar at baseline on important characteristics i.e. demographics, risk factors, co-morbid conditions? [score 0=no/not reported, 1=partially matched/possibly similar, 2= yes] |
| Q-9 | Were inclusion and exclusion criteria prespecified and applied uniformly to all participants? [score 0=no/not reported, 1=partially stated/applied, 2= yes] |
| Q-10 | Was the exposure clearly defined and implemented – the intervention? [score 0=no/not reported/not defined, 1=partially defined/mentioned, 2= yes] |
| Q-11 | If present was the control or comparison arm appropriate? [score 0=unlikely 1=possibly, 2=probable] |
| Q-12 | Was the outcome clearly defined and implemented? [score 0=no/not reported/not defined, 1=partially defined/mentioned, 2= yes] |
| Q-13 | How reliable were the methods used for the determination of outcome (pancreatic functions)? [score 0 = poor, 1=ok, 2=good] |
| Q-14 | Was sample size justified - , power description, or variance and effect estimates provided? [score 0 = poor, 1=ok, 2=good] |
| Q-15 | Was the sample size big enough for main comparison or association being tested (or the one we are interested in for this paper if not the main objective of the paper)? [score 0=unlikely, 1=possibly, 2= yes] |
| Q-16 | Did the study examine any dose response effect? [score 0=unlikely, 1=partially, 2= yes] |
| Q-17 | Was the duration of exposure assessed? [score 0=no/not reported, 1=partially assessed, 2= yes] |
| Q-18 | Were the outcome assessors blinded to the exposure status of participants? [score 0=unlikely, 1=possibly, 2= yes] |
| Q-19 | Were the study participants and researchers blinded to treatment group assignment? [score 0=No, 1=participants or investigators, 2= both] |
| Q-20 | Was the method of analysis appropriate? [score 0=no/not reported, 1=possibly, 2= yes] |
| Q-21 | If NOT randomised - were key potential confounding variables measured and adjusted statistically for their impact on the relationship between exposure(s) and outcome(s)? [score 0=unlikely, 1=partially, 2= yes] |
| Q-22 | If RCT – were all randomized participants analyzed in the group to which they were originally assigned, i.e., did they use an intention-to-treat analysis? [score 0=unlikely, 1=partially, 2= yes] |
| CD, cannot be determined (if CD, point 0)., NA, not applicable (not account in final scoring), NR, not reported (if NR, point 0)., N, no (if N/unlikely, point 0), PA, partially/possibly answered (if PA, point 1), Y, yes (if Y, point 2). ≥ 80% = High; 60-79% = Medium; , 45-59% = Low; <45% = V. Low | |

**Supplementary table 3.2 Quality assessment score for the intervention/randomized control trial study designs to assess quality/strength of the evidence.**

|  | **Q-1** | **Q-2** | **Q-3** | **Q-4** | **Q-5** | **Q-6** | **Q-7** | **Q-8** | **Q-9** | **Q-10** | **Q-11** | **Q-12** | **Q-13** | **Q-14** | **Q-15** | **Q-16** | **Q-17** | **Q-18** | **Q-19** | **Q-20** | **Q-21** | **Q-22** | **Rating** |
| --- | --- | --- | --- | --- | --- | --- | --- | --- | --- | --- | --- | --- | --- | --- | --- | --- | --- | --- | --- | --- | --- | --- | --- |
| **Bartels, 2017** | 2 | 2 | 2 | 2 | 1 | 1 | 2 | 2 | 2 | 2 | 1 | 2 | 2 | 2 | 2 | 2 | 2 | 0 | 1 | 2 | NA | 2 | **85.7** |
| **Becker, 1975** | 1 | 1 | 1 | 2 | 0 | 0 | 0 | 2 | 2 | 2 | 1 | 2 | 2 | 0 | 0 | 2 | 2 | 0 | 0 | 2 | NA | 1 | **54.8** |
| **Mehta, 1984** | 2 | 1 | 1 | 2 | 0 | NA | NA | 1 | 1 | 2 | 1 | 2 | 1 | 0 | 0 | 1 | 2 | 1 | 0 | 1 | 0 | 1 | **50.0** |
| **Robinson, 1982** | 2 | 1 | 1 | 0 | 0 | NA | NA | 1 | 1 | 2 | 1 | 2 | 2 | 0 | 0 | 0 | 2 | 0 | 0 | 1 | 0 | 1 | **42.5** |
| **Sauniere, 1988** | 2 | 2 | 2 | 2 | 0 | 0 | 0 | 1 | 1 | 2 | 2 | 2 | 2 | 0 | 0 | 0 | 2 | 0 | 0 | 1 | NA | 2 | **54.8** |
| **Thompson, 1952** | 1 | 1 | 1 | 1 | 0 | NA | NA | 0 | 2 | 1 | 1 | 1 | 2 | 0 | 0 | 2 | 2 | 0 | 0 | 0 | 0 | 2 | **42.5** |

**Supplementary table 3.3 Quality assessment tools and score for the case-control trial study designs to assess quality/strength of the evidence.**

|  | **Assessment tools** |
| --- | --- |
| Q-1 | Was the research question or objective clearly stated? [score 0=not reported/no, 1=partially stated, 2=yes] |
| Q-2 | Was the study design clearly stated? [score 0=not reported/no, 1=partially stated, 2=yes] |
| Q-3 | Was the study population clearly defined? [score 0=not reported/no, 1=partially stated, 2=yes] |
| Q-4 | If a case-control study - were controls selected or recruited from the same or similar population (including the same timeframe)? [score 0=no/not reported, 1=partially performed, 2=recruited from similar population using same timeframe] |
| Q-5 | Was the sampling method/s clearly defined and appropriate for the question being asked? [score 0=no/not reported, 1=partially defined, 2=yes] |
| Q-6 | Were inclusion and exclusion criteria prespecified and applied uniformly to all participants? [score 0=no/not reported, 1=partially stated/applied, 2= yes] |
| Q-7 | If a case-control study - were the processes for identifying or selecting cases and controls clearly defined? Were cases clearly defined and differentiated from controls? [score 0=no/not reported/not defined, 1=partially reported/mentioned, 2= yes] |
| Q-8 | Was the exposure clearly defined and implemented (malnutrition, or exposure to famine)? [score 0=no/not reported/not defined, 1=partially defined/mentioned, 2= yes] |
| Q-9 | If a case-control study - How probable that the exposure/risk occurred prior to the development of the condition or event that defined a participant as a case? [score 0=unlikely 1=possibly, 2=probable] |
| Q-10 | Was there an appropriate control/comparison group? [score 0=unlikely 1=possibly, 2=probable] |
| Q-11 | Was the outcome clearly defined and implemented? [score 0=no/not reported/not defined, 1=partially defined/mentioned, 2= yes] |
| Q-12 | How reliable were the methods used for the determination of outcome (pancreatic functions)? [score 0 = poor, 1=ok, 2=good] |
| Q-13 | Was sample size justified - , power description, or variance and effect estimates provided? [score 0 = poor, 1=ok, 2=good] |
| Q-14 | Was the sample size big enough for main comparison or association being tested (or the one we are interested in for this paper if not the main objective of the paper)? [score 0=unlikely, 1=possibly, 2= yes] |
| Q-15 | Did the study examine any dose response effect? [score 0=unlikely, 1=partially, 2= yes] |
| Q-16 | Was the duration of exposure assessed? [score 0=no/not reported, 1=partially assessed, 2= yes] |
| Q-17 | Were the outcome assessors blinded to the exposure status of participants? [score 0=unlikely, 1=possibly, 2= yes] |
| Q-18 | Was the method of analysis appropriate? [score 0=no/not reported, 1=possibly, 2= yes] |
| Q-19 | Were key potential confounding variables measured and adjusted statistically for their impact on the relationship between exposure(s) and outcome(s)? [score 0=unlikely, 1=partially, 2= yes] |
| Q-20 | If a case-control study – if matching was used, did the study account for matching during the study analysis? Was the matching appropriate (i.e. not matched on the exposures of interest) [score 0=poor, 1=ok, 2= good] |
| CD, cannot be determined (if CD, point 0)., NA, not applicable (not account in final scoring), NR, not reported (if NR, point 0)., N, no (if N/unlikely, point 0), PA, partially/possibly answered (if PA, point 1), Y, yes (if Y, point 2), ≥ 80% = High; 60-79% = Medium; 45-59% = Low; <45% = V. Low | |

**Supplementary table 3.3 Quality assessment tools and score for the case-control trial study designs to assess quality/strength of the evidence.**

|  | **Q-1** | **Q-2** | **Q-3** | **Q-4** | **Q-5** | **Q-6** | **Q-7** | **Q-8** | **Q-9** | **Q-10** | **Q-11** | **Q-12** | **Q-13** | **Q-14** | **Q-15** | **Q-16** | **Q-17** | **Q-18** | **Q-19** | **Q-20** | **Rating** |
| --- | --- | --- | --- | --- | --- | --- | --- | --- | --- | --- | --- | --- | --- | --- | --- | --- | --- | --- | --- | --- | --- |
| **Fransis-Emmanuel, 2014** | 2 | 2 | 2 | 2 | 1 | 2 | 2 | 2 | 1 | 2 | 2 | 2 | 0 | 1 | 2 | 1 | 0 | 2 | 2 | 0 | **75.0** |
| **Gonzalez-Barranco, 2003** | 2 | 2 | 2 | 2 | 1 | 1 | 2 | 1 | 2 | 1 | 2 | 2 | 0 | 1 | 2 | 1 | 0 | 2 | 2 | NA | **70.0** |
| **Sathiaraj, 2010** | 2 | 2 | 2 | 2 | 1 | 2 | 1 | 2 | 1 | 2 | 1 | 1 | 0 | 1 | 0 | 1 | 1 | 2 | 0 | NA | **63.2** |
